# Germ cell apoptosis is critical to maintain *Caenorhabditis elegans* offspring viability in stressful environments

**DOI:** 10.1371/journal.pone.0260573

**Published:** 2021-12-08

**Authors:** Sarah Fausett, Nausicaa Poullet, Clotilde Gimond, Anne Vielle, Michele Bellone, Christian Braendle

**Affiliations:** Université Côte d’Azur, CNRS, Inserm, IBV, Nice, France; INSERM U869, FRANCE

## Abstract

Maintaining reproduction in highly variable, often stressful, environments is an essential challenge for all organisms. Even transient exposure to mild environmental stress may directly damage germ cells or simply tax the physiology of an individual, making it difficult to produce quality gametes. In *Caenorhabditis elegans*, a large fraction of germ cells acts as nurse cells, supporting developing oocytes before eventually undergoing so-called physiological germ cell apoptosis. Although *C*. *elegans* apoptosis has been extensively studied, little is known about how germline apoptosis is influenced by ecologically relevant environmental stress. Moreover, it remains unclear to what extent germline apoptosis contributes to maintaining oocyte quality, and thus offspring viability, in such conditions. Here we show that exposure to diverse environmental stressors, likely occurring in the natural *C*. *elegans* habitat (starvation, ethanol, acid, and mild oxidative stress), increases germline apoptosis, consistent with previous reports on stress-induced apoptosis. Using loss-of-function mutant alleles of *ced-3* and *ced-4*, we demonstrate that eliminating the core apoptotic machinery strongly reduces embryonic survival when mothers are exposed to such environmental stressors during early adult life. In contrast, mutations in *ced-9* and *egl-1* that primarily block apoptosis in the soma but not in the germline, did not exhibit such reduced embryonic survival under environmental stress. Therefore, *C*. *elegans* germ cell apoptosis plays an essential role in maintaining offspring fitness in adverse environments. Finally, we show that *ced-3* and *ced-4* mutants exhibit concomitant decreases in embryo size and changes in embryo shape when mothers are exposed to environmental stress. These observations may indicate inadequate oocyte provisioning due to the absence of germ cell apoptosis. Taken together, our results show that the central genes of the apoptosis pathway play a key role in maintaining gamete quality, and thus offspring fitness, under ecologically relevant environmental conditions.

## Introduction

Maintaining reproduction in stressful environments is an essential challenge for all organisms. Adverse conditions, both transient and long-term, can strongly affect male or female gamete quality and production and, thus, impact final reproductive output and fitness. However, despite a multitude of molecular mechanisms known to be involved in germline integrity, it is often unclear how their action translates into the maintenance of gamete quality, and hence offspring fitness, under ecologically relevant environmental stress.

The nematode *Caenorhabditis elegans* is one of the major models to study germ cell development and the genetics of gametogenesis [1–3], including germline integrity in response to stress [4, 5]. The adult *C*. *elegans* hermaphrodite gonad consists of two identical U-shaped tubes, connected to the central uterus, each containing about 1000 germ cells in replete standard laboratory conditions [2]. At the most distal tip of each gonad arm, the germ stem cell (GSC) nuclei reside in a syncytium enwrapped by a somatic distal tip cell (DTC) that maintains their stemness and responds to internal and external cues to modulate GSC proliferation and differentiation [3]. As the nuclei proliferate, they pass proximally, away from the GSC niche, and begin meiosis. Initially, during the early L4 larval stage, all meiotic germ cells develop into sperm. This is followed by an irreversible switch to oogenesis that continues throughout adulthood [1].

**Fig 1:**
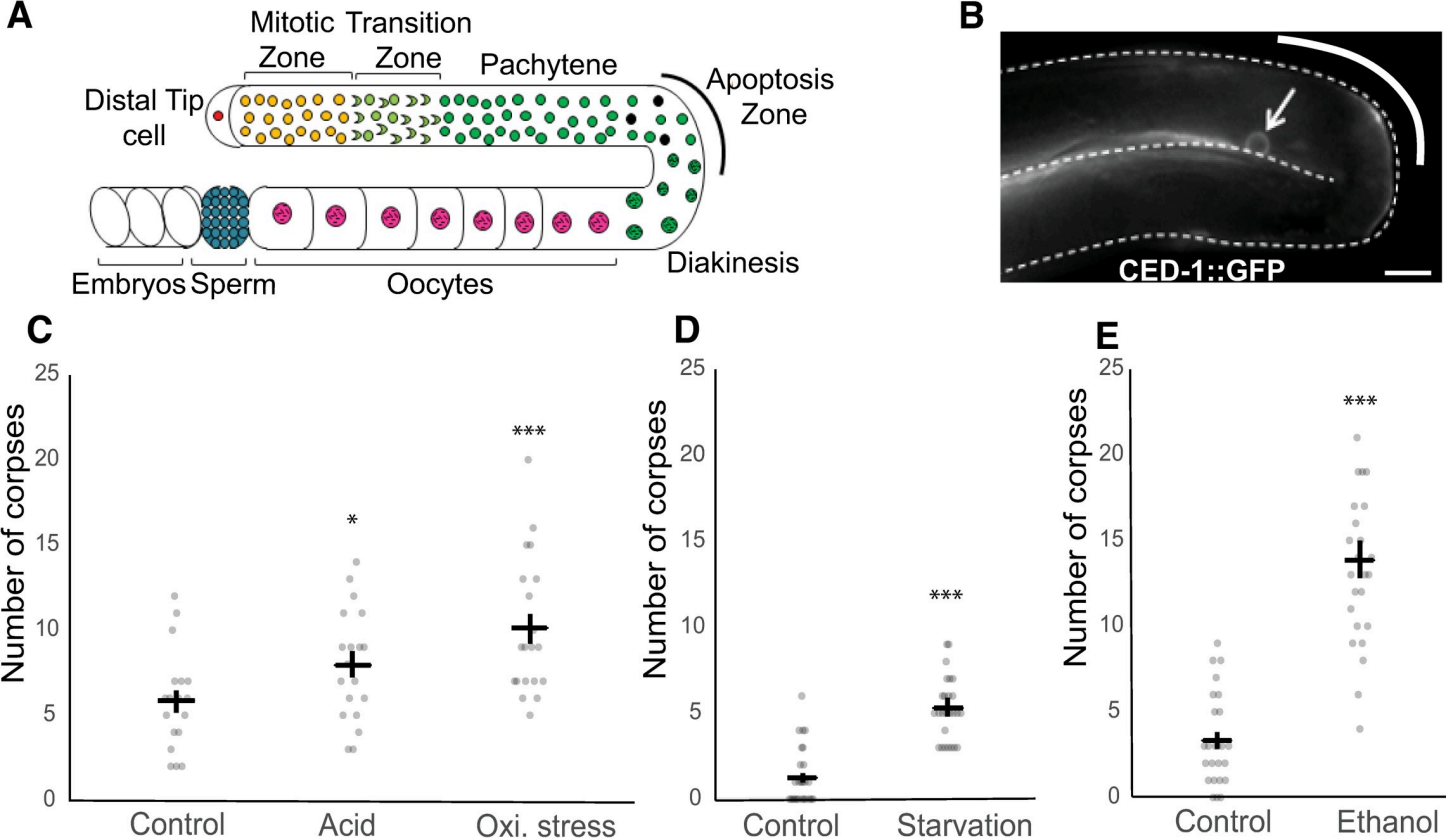
Diverse environmental stress conditions increase steady state apoptotic corpses in the germline. (A) Diagram of a single hermaphrodite gonad arm. Germ cells are born in the germ stem cell niche (Mitotic Zone). As they are displaced by the proliferation of germ cells in this region, they exit the niche, enter the Transition Zone and commit to meiosis. In late pachytene, a subset of germ cells undergoes physiological apoptosis while others begin to grow larger as they are provisioned via cytoplasmic streaming from the rachis and receptor-mediated endocytosis from the pseudocoelom [10, 17]. As meiosis 1 completes, large oocytes form a line, enter the spermatheca one at a time during ovulation, and are fertilized. Fertilized embryos develop in the uterus until they are laid. (B) A fluorescent micrograph of the germline loop region of an adult hermaphrodite (L4+24h) in control conditions. The arrow indicates an apoptotic corpse expressing CED-1::GFP. Scale bar: 20μm. (C-D) Number of germ cell corpses per gonad arm after (C) acid (HCl) or oxidative stress (paraquat) exposure, and (D) starvation or (E) ethanol exposure. Black bars indicate estimated marginal means +/- standard error. (* p = 0.01–0.05, ** p = 0.001–0.01, *** p < 0.001; comparisons are all to control for each condition). For statistical models (negative binomial regression) and results, see S1 Table.

A notable feature of oogenesis in *C*. *elegans* is that about half of the oogenic germ cells are eliminated by apoptosis [3]. Such germ cell apoptosis, also termed physiological germ cell apoptosis, occurs in the germline loop region where the germ cells destined to become oocytes begin to cellularize and grow substantially larger after the pachytene stage of prophase II (Fig 1A). Physiological germ cell apoptosis is initiated by the core apoptotic machinery. This core pathway relies on a protein interaction cascade involving the anti-apoptotic protein CED-9, which inhibits the caspase activator CED-4 and the caspase CED-3 [6]. However, its mechanism is different from somatic cell apoptosis in that it does not rely on the pro-apoptotic BH3-only protein, EGL-1. A key regulator of physiological germline apoptosis is the RAS/MAPK signaling pathway; no apoptosis occurs when the RAS/MAPK pathway is blocked, whereas excessive apoptosis occurs when it is over-activated [3, 7]. This pathway is also important for the increased apoptosis seen after osmotic, oxidative, and heat stress [8]. Meanwhile, LIN-35 modulates germ cell apoptosis in response to starvation stress [9]. There is now good evidence supporting the hypothesis that the germ cells destined to undergo physiological apoptosis act as nurse cells for the developing oocytes, supplying cytoplasm to the growing oocytes through cytoplasmic streaming [10–13]. The clearest experimental evidence that physiological germ cell apoptosis contributes to the maintenance of oocyte quality has been shown during reproductive aging, as blocking apoptosis by mutation of *ced-3* or *ced-4* leads to reduced oocyte size in old mothers and increased embryonic death of their offspring [11]. In addition, although lacking conclusive experimental evidence, apoptosis may further contribute to germline integrity by eliminating damaged and defective oocytes [3, 14, 15]. Finally, the core apoptotic machinery also functions in diverse non-apoptotic processes, including cell differentiation, reprogramming and stress responses [reviewed in 16].

A central question is how *C*. *elegans* germ cell apoptosis contributes to the maintenance of gamete quality in response to environmental stress. Different types of apoptosis can be upregulated by deleterious environmental factors, including pathogens, heat stress, oxidative stress or starvation [8, 18–21] as well as genotoxic stressors, such as DNA damage [14]. Although it is now clear that diverse acute stressors increase germ cell apoptosis, these studies have rarely examined germ cell apoptosis in response to environmental challenges that may be closer to what *C*. *elegans* experiences in the wild [8, 12, 14, 19, 20, 22]. *C*. *elegans*, in its natural habitat primarily consisting of decomposing, microbe-rich plant matter [23, 24], faces a multitude of potentially stressful environmental factors, including food deprivation as well as various fermentation and decomposition products that affect local ethanol concentrations, acidity, and oxidative species. Furthermore, while many studies have contributed greatly to our understanding of germline molecular and cellular biology, apoptosis and other germline processes are often examined in isolation, without relating them to final reproductive output [4, 5, 25–27]. Thus, the question of how *C*. *elegans* germline apoptosis acts specifically to maintain reproductive output under ecologically relevant environmental variation deserves further study.

Here we build on several key studies [8, 11, 19] to present evidence that the core machinery driving physiological germ cell apoptosis is critical, specifically in young mothers, to preserve oocyte quality and fertility in ecologically relevant stress conditions (starvation, ethanol, acidic stress, and oxidative stress). We further demonstrate that, as in the case of reproductive aging [11], germline apoptosis generally supports the production of well-formed and well-provisioned oocytes, and that this is especially important in adverse environmental conditions.

## Results

### Diverse environmental stress conditions increase steady state apoptotic corpses in the germline

To assess the effects of diverse, ecologically relevant environmental stressors on germline apoptosis, we exposed young adult hermaphrodites bearing *ced-1*::GFP (MD701) [66] to 20h liquid starvation or NGM plates supplemented with HCl (0.01M, 12h), or paraquat (0.5mM 12h). We also exposed worms during the development from the L1 stage to the adult stage (L4+24h) to 0.32M ethanol-supplemented NGM plates [65]. GFP-positive apoptotic corpses in the germline were counted immediately after stress treatment (Fig 1B). Among the stress treatments, we found that all four increased the number of GFP-positive apoptotic corpses, with the greatest increases seen after ethanol and paraquat treatment (Fig 1C–1E), indicating an increase in germ cell apoptosis or possibly a delay in apoptotic corpse engulfment. Given that germ cell number in both mitotic and meiotic regions are strongly reduced under these and other stress conditions [28], the observed increase in GFP-positive cells does not simply represent an overall increase in germ cells.

### CED-3 maintains embryonic viability in stressful conditions

**Fig 2:**
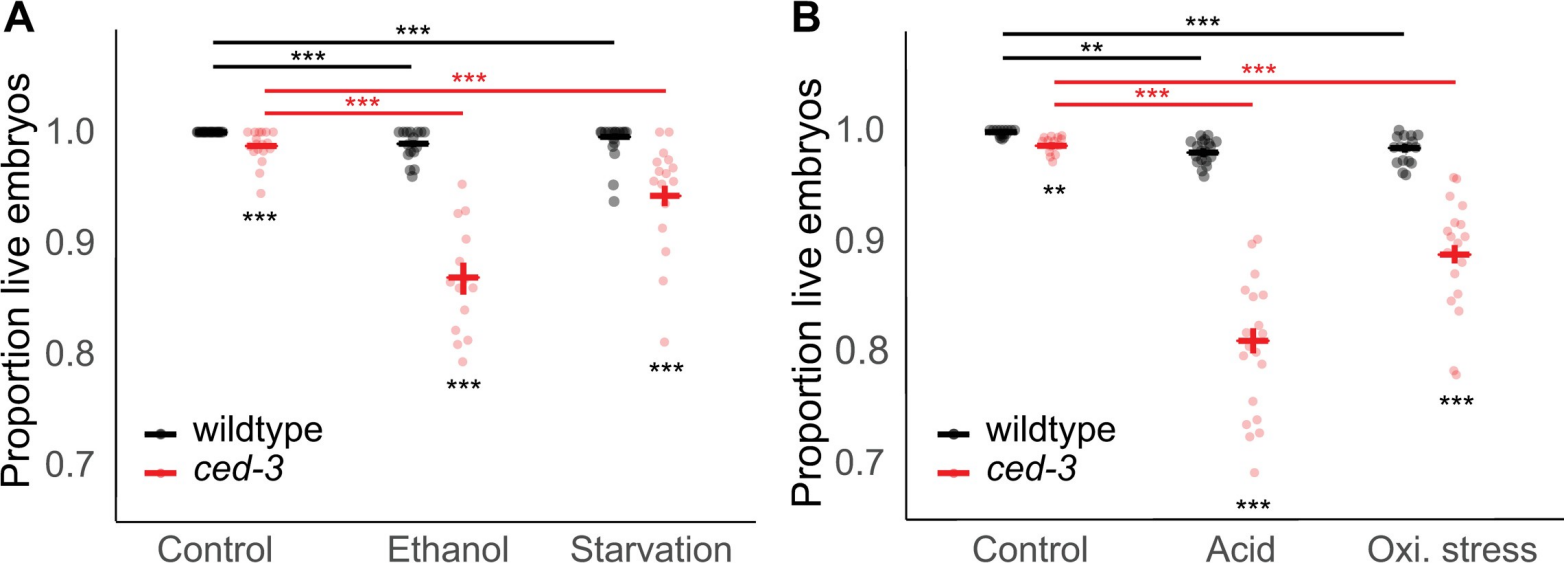
Apoptosis maintains embryonic viability in stressful conditions. (A) Embryonic survival (living L1s / (living L1s + dead embryos)) of wildtype (N2) and *ced-3(n718)* animals grown on control NGM plates, ethanol supplemented plates, or subjected to liquid starvation for 20h from L4+20h. (B) Survival of embryos (living L1s / (living L1s + dead embryos)) laid between L4+24h and L4+72h from wildtype (N2) and *ced-3(n718)* animals exposed to HCl or paraquat supplemented plates or control NGM plates for 12 hours at L4+12h. Bold blue or red bars indicate estimated marginal means +/- standard error. (* p = 0.01–0.05, ** p = 0.001–0.01, *** p < 0.001; black asterisks indicate comparisons within environment while colored asterisks indicate comparisons within genotype). For statistical models (betabinomial regression) and results, see S2 Table.

The mutant strain *ced-3(n718)* [29] had previously been shown to exhibit slightly increased embryonic lethality relative to the wild type strictly due to maternal (oocyte) effects [11]. More recently, Carranza-Garcia and Navarro demonstrated that *ced-3* is also important to maintain oocyte quality during the oogenic germline starvation response after five days of starvation from the L4 larval stage [19], such that embryonic lethality is exacerbated in mothers lacking functional *ced-3*. To build on these findings, we examined the effects of environmental stress conditions on embryonic survival. In wildtype mothers, ethanol and starvation stress only slightly reduced embryonic survival by 1.1% and 0.3%, respectively. However, in mothers lacking functional *ced-3*, embryonic survival was reduced by 12% under ethanol stress and 4.5% under starvation (Fig 2A and S2D and S2F Table). Similarly, acid and oxidative stress reduced survival of embryos from wildtype mothers by 1.8% and 1.5%, respectively, but from *ced-3(n718)* mothers by 17.6% and 9.7%, respectively (Fig 2B and S2A and S2C Table). We also observed that *ced-3(n718)* exacerbates the tendency of these environments to reduce the total number of embryos produced from L4+24h to L4+72h (S1 Fig and S10 Table).

### Stress-induced embryonic lethality observed in *ced-3(n718)* mutants is due to reduced oocyte quality

**Fig 3:**
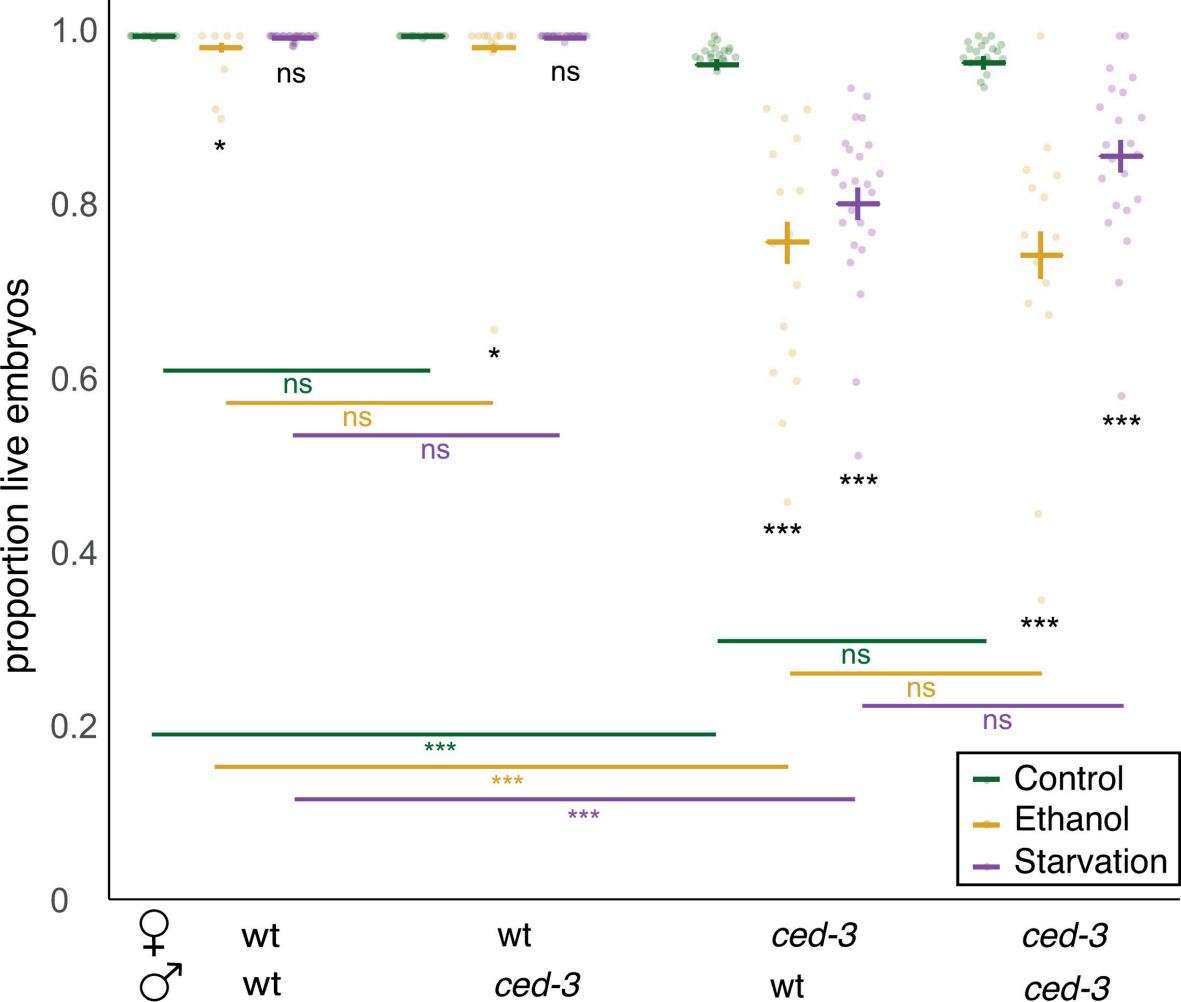
Stress-induced embryonic lethality observed in *ced-3(n718)* mutants is due to reduced oocyte quality. Virgin *fog-2* females with *ced-3(n718)* or without were subjected to ethanol or starvation treatment and then allowed to mate with untreated males of the indicated genotype on control NGM plates and to lay eggs. Embryonic survival rate was calculated for all embryos laid for 48h. Bold blue or red bars indicate estimated marginal means +/- standard error. (* p = 0.01–0.05, ** p = 0.001–0.01, *** p < 0.001). For statistical models (betabinomial regression) and results, see S3 Table.

To test whether the reductions in embryonic survival of *ced-3* mutants were strictly maternal, and thus due to reduced oocyte quality, we quantified embryonic lethality after ethanol and starvation treatments using the double mutant strain, *fog-2(q71);ced-3(n718)*, with the *fog-2 (q71)* mutation abolishing self-sperm production [30]. Reciprocal crosses were performed between *fog-2(q71);ced-3(n718)* females and *fog-2(q71);ced-3(n718)* males. This allowed testing whether heterozygous embryos (maternal *ced-3*, paternal *wt*) show the same, increased embryonic lethality as *ced-3(n718)* homozygous embryos. Using this method, we found that maternal *ced-3(n718)* was sufficient to significantly decrease embryo survival in these two stress conditions, and the paternal genotype was largely irrelevant (Fig 3 and S3 Table). These results show that *ced-3* in the mother has an essential role in maintaining oocyte quality and resulting offspring viability in stressful environments. These results are in agreement with previous studies showing that physiological germ cell apoptosis is required for the maintenance of oocyte quality during long-term starvation [11, 19] as well as in aging individuals [11, 19].

### Multiple *ced-3* and *ced-4 lf* alleles reduce embryonic viability under ethanol and starvation stress

**Fig 4:**
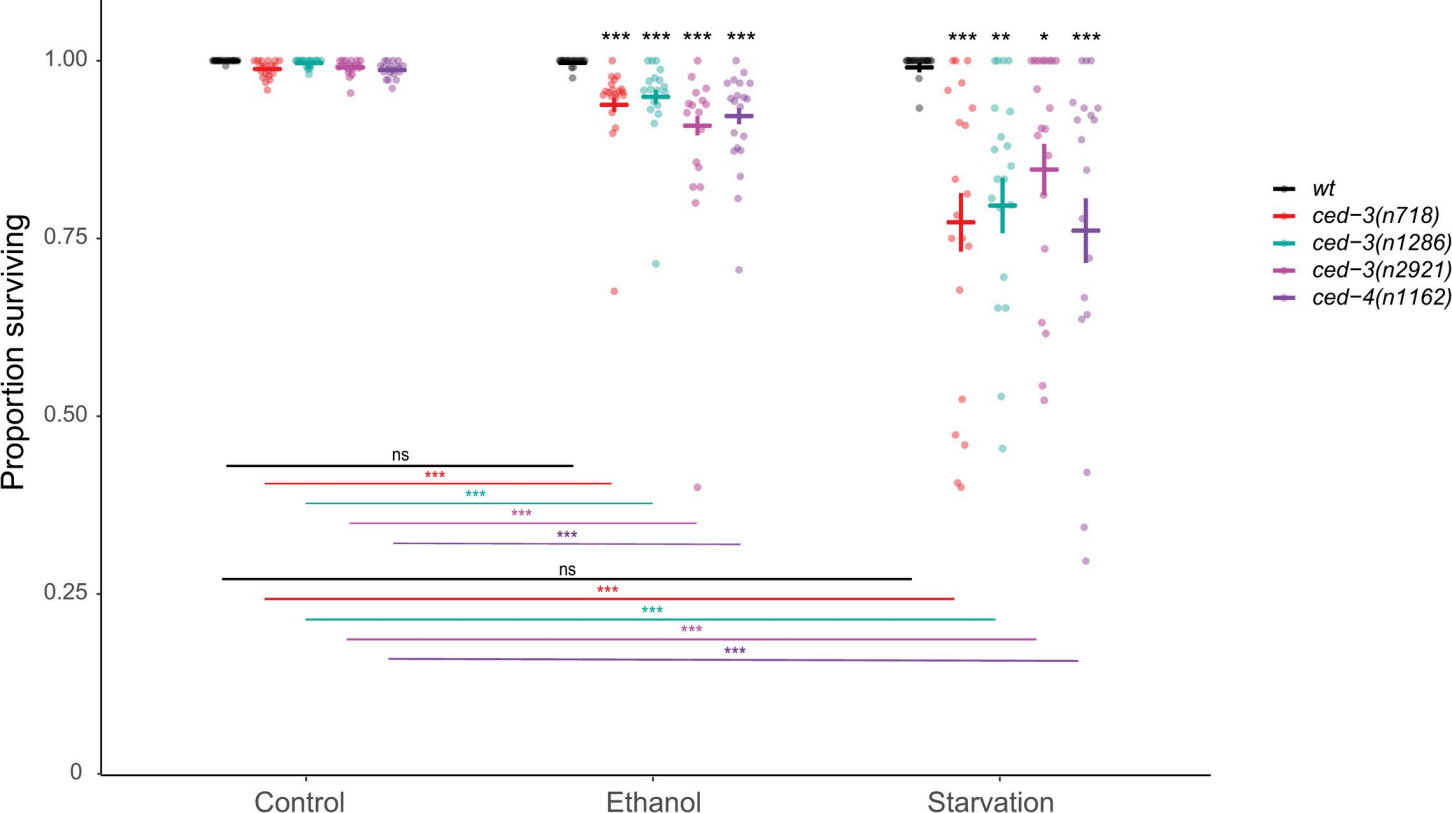
Multiple *ced-3* and *ced-4 lf* alleles reduce embryonic viability under ethanol and starvation stress. Embryonic survival (living L1s / (living L1s + dead embryos)) in wildtype (N2), *ced-3(n718)*, *ced-3(n1286)*, *ced-3(n2921)*, and *ced-4(n1162)* animals grown on control NGM plates, ethanol supplemented plates, or subjected to liquid starvation for 20h from L4+20h. Bold bars indicate estimated marginal means +/- standard error. (* p = 0.01–0.05, ** p = 0.001–0.01, *** p < 0.001). For statistical models (betabinomial regression) and results, see S4 Table.

To validate our findings based on the analysis of the single *ced-3(n718)* allele, we next quantified embryonic survival under ethanol and starvation stress using multiple alleles of *ced-3* and *ced-4*. Embryonic survival did not differ between the genotypes under control conditions, but all *ced-3* and *ced-4 lf* alleles reduced embryonic viability after exposure to ethanol or starvation stress with a significant genotype x environment effect (Fig 4). These results confirm that the core apoptotic machinery driving physiological germ cell apoptosis is critical for the maintenance of embryonic viability in stressful environments.

### Mutations that abolish or reduce apoptosis in the soma but not the germline are dispensable for embryonic survival under environmental stress

**Fig 5:**
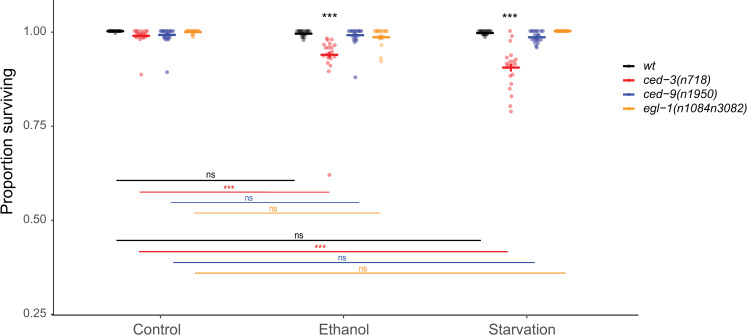
*ced-9(gf)* and *egl-1(lf)* do not reduce embryonic survival under ethanol or starvation stress. Embryonic survival (living L1s / (living L1s + dead embryos)) in wildtype (N2), *ced-3(n718)*, *ced-9(n1950gf)*, *egl-1(n1084n3082)* animals grown on control NGM plates, ethanol supplemented plates, or subjected to liquid starvation for 20h from L4+20h. Bold bars indicate estimated marginal means +/- standard error. (* p = 0.01–0.05, ** p = 0.001–0.01, *** p < 0.001; black asterisks indicate comparisons within environment while colored asterisks indicate comparisons within genotype). For statistical models (betabinomial regression) and results, see S5 Table.

Loss-of-function mutations in *egl-1*, *ced-13*, *cep-1* or gain-of-function mutations in *ced-9* are known to inhibit germ cell apoptosis in response to DNA damage stress, but they have no or little effect on physiological germ cell apoptosis [3, 14, 25, 31–33]. Furthermore, somatic cell apoptosis, but not physiological germ cell apoptosis is inhibited by loss-of-function mutations in *egl-1* or gain-of-function mutations in *ced-9* [3, 34]. We found that, under ethanol and starvation stress, *egl-1(lf)* and *ced-9(gf)* mutants had no effect on embryonic lethality, whereas the *n718* loss-of-function mutation in *ced-3* severely reduced embryonic viability (Fig 5). We conclude that reduced embryo survival under environmental stress is due to loss of germline physiological apoptosis but not dependent on somatic cell apoptosis nor the DNA-damage response pathway.

### The decrease in embryonic survival after stress correlates with a decrease in oocyte and embryo size

**Fig 6:**
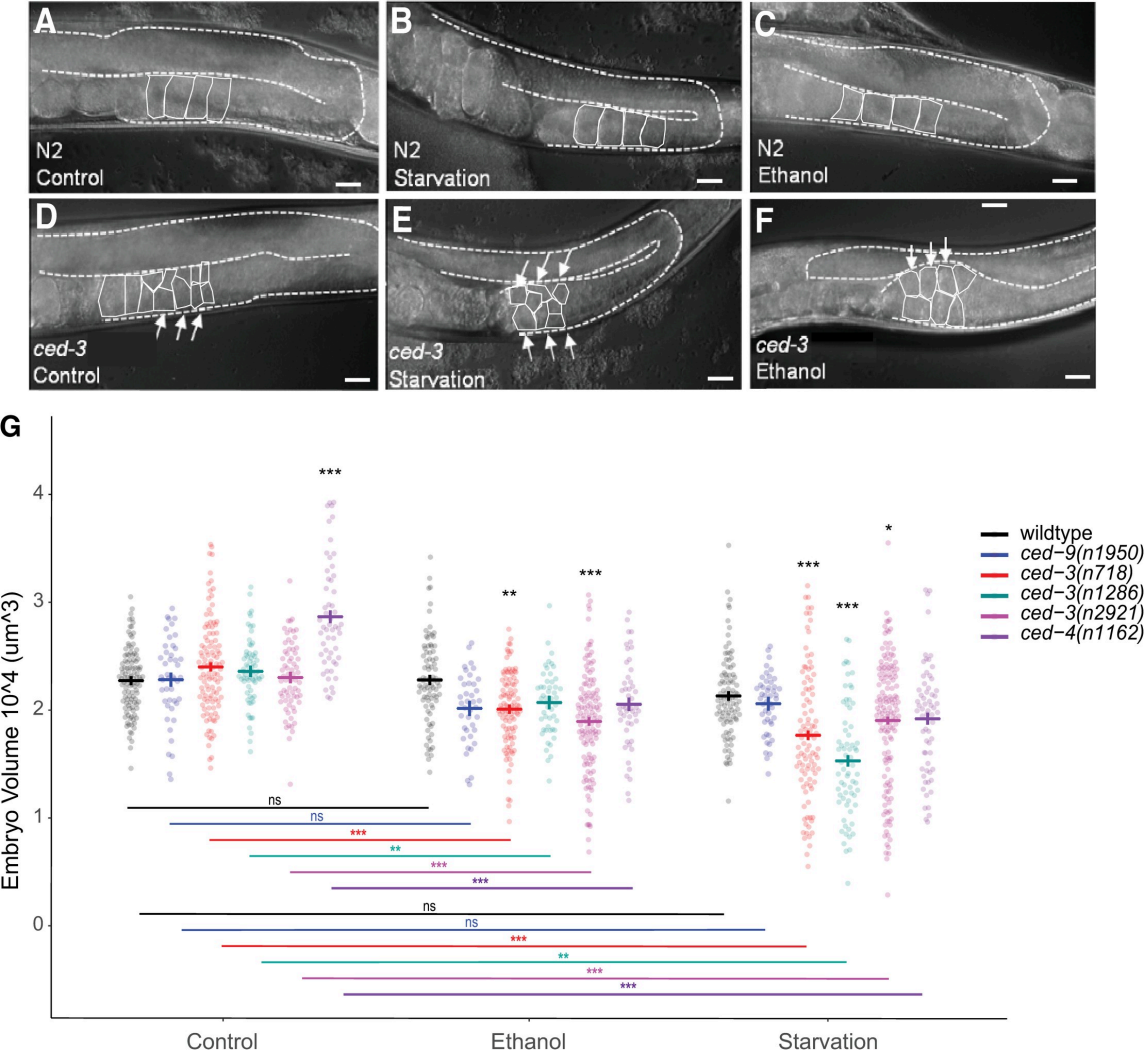
The decrease in embryonic survival after stress coincides with a decrease in oocyte and embryo size. (A-F) DIC images of individual gonad arms in the wildtype strain N2 (A-C) and the mutant *ced-3(n718)* (D-F) adult hermaphrodites grown on control NGM plates (A, D), subjected to 20h liquid starvation at L4+20h (B, E), or grown on ethanol-supplemented plates (0.32M) from L1 –L4+24h (C, F). Proximal oocytes are outlined in solid white for clarity. Dashed lines delineate the gonad. Scale bars: 20μm. (G) Size (volume) of embryos produced by N2 and mutant adults in control, ethanol, and starvation conditions. Bold blue or red bars indicate estimated marginal means +/- standard error. (* p = 0.01–0.05, ** p = 0.001–0.01, *** p < 0.001; black asterisks indicate comparisons within environment while colored asterisks indicate comparisons within genotype). For statistical models (standard linear regression) and results, see S6 Table.

Stress-induced decline of oocyte quality in apoptosis-defective mutants may manifest itself through multiple causes; however, it has been shown that one specific oocyte characteristic may be linked to this decline: oocyte size. Andux and Ellis (2008) reported a significant reduction of oocyte size in *ced-3* mutants, suggesting that germ cell apoptosis is required for oocyte provisioning and growth [11]. In young adults reared under both control and stress conditions, we observed that the oocytes of *ced-3(n718)* mutants were smaller and spread over several layers of the germline, whereas in wildtype, each occupied an entire section of the proximal germline (Fig 6A–6F). Given that oocyte size is the principal determinant of embryo size [35, 36], we measured the volume of laid embryos of *ced-3 and ced-4* mutants as compared to wild type and *ced-9(gf)* animals in control versus stress conditions (Fig 6G). *ced-3(lf)* or *ced-4(lf)*, but not wildtype or *ced-9(gf)*, had significantly reduced embryo volume under starvation or ethanol as compared to control conditions (Fig 6G). Therefore, significant reduction of embryo size only occurs when specifically blocking germ cell apoptosis. Since embryo size is directly linked oocyte size, and thus to oocyte resource provisioning [10, 11, 37], our observation that embryo survival of *ced-3* and *ced-4* mutants is reduced under environmental stress (Fig 3) could, thus, be explained by perturbed, insufficient oocyte provisioning in the absence of germ cell apoptosis. In addition, environmental stress increased the variability of embryo size, in particular, in the three *ced-3* mutant genotypes under starvation (Fig 6G), suggesting that germ cell apoptosis is also required for precise, reproducible oocyte provisioning.

### The relationship between egg volume and egg shape is altered in *ced-3* and *ced-4* mutants under environmental stress

**Fig 7:**
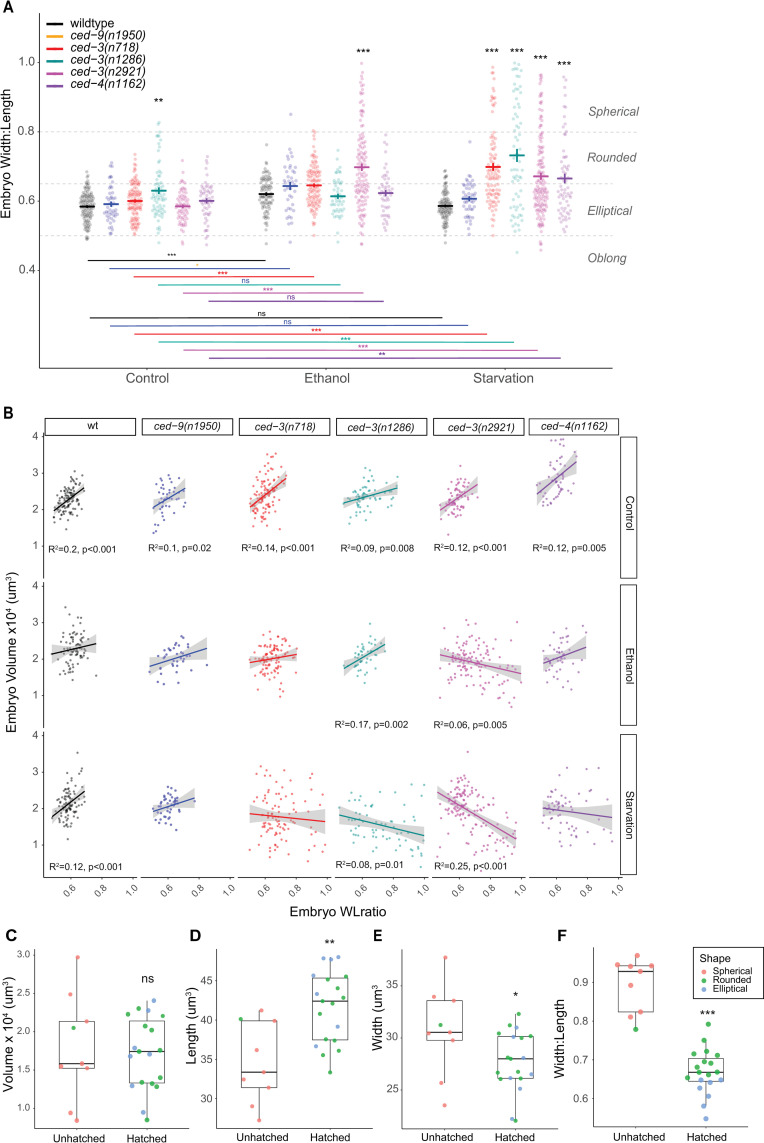
The relationship between egg volume and egg shape is altered in germ cell apoptosis mutants under environmental stress. (A) Shape (width:length ratio of embryos produced by N2 and mutant adults in control, ethanol, and starvation conditions. Bold blue or red bars indicate estimated marginal means +/- standard error. (* p = 0.01–0.05, ** p = 0.001–0.01, *** p < 0.001; black asterisks indicate comparisons within environment while colored asterisks indicate comparisons within genotype). For statistical models (standard linear regression) and results, see S7 Table. (B) Correlations between embryo volume and embryo shape among all genotypes and environments based on linear regression. Adjusted R^2^ and p-values are reported here only for significant correlations. See S8 Table for linear regression summaries. (C-F) *ced-3(n718)* embryos subjected to starvation stress were monitored for hatching over 48h. Volume (C), length (D), width (E), and width:length ratio (F) are shown for embryos that hatched or did not hatch. Colors indicate embryo shape based on the thresholds indicated in panel A (Spherical: >0.8, Rounded: 0.65–0.8, Elliptical: 0.5–0.65, Oblong: <0.5). (* p = 0.01–0.05, ** p = 0.001–0.01, *** p < 0.001). See S9 Table for detailed statistics.

In the course of conducting the experiments described above, we also noticed a change in the shape of the embryos under stress conditions. In particular, embryos laid by starved mothers of *ced-3* and *ced-4* mutants appeared spherical in addition to being smaller. We, therefore, quantified embryo shape by calculating the width:length ratio (WLratio) and asked whether embryo shape might also correlate with higher embryonic lethality regardless of volume. We found that among genotype-environment combinations with high embryonic lethality, embryos tended to be rounder and even close to spherical (WLrate >0.8). This was particularly evident in starvation conditions (Fig 7A). A rounder shape might simply be the result of reduced embryo volume, in which case we should see a correlation between the WLratio and volume. However, when we examined the correlation between the two variables, we found that the relationship is more complex (Fig 7B). In control conditions, for wildtype and all tested mutants, embryo volume increased with the WLratio, indicating that larger embryos were somewhat rounder (Fig 7B). This relationship was also largely maintained for wildtype and *ced-9* in stress conditions. However, in *ced-3* and *ced-4* mutants under stress, the relationship between embryo volume and shape broke down and was, in some cases, even reversed, i.e. the smallest embryos were the roundest (Fig 7B). To evaluate which embryo size measure might be the most reliable predictor of embryonic survival, we examined *ced-3(718)* mutants, which have high embryonic lethality under starvation conditions. We measured the embryo length and width of a subset of embryos laid by *ced-3* mothers exposed to starvation and then monitored hatching of individual embryos over the next 48 hours. In this small batch of embryos, volume did not differ between hatched and unhatched embryos (Fig 7C); however, embryo length, width and WLratio (shape) were all significantly different between hatched and unhatched embryos (Fig 7C–7F). While overall effects of environmental stress on embryo survival (Fig 6G) and embryo volume coincide for all tested *ced-3* and *ced-4* mutants (Fig 6A), differences in embryo volume alone seem to be insufficient to predict differences in embryonic survival.

## Discussion

Consistent with previous reports using different protocols and experimental environments [8, 9, 18], we show here that exposure to various environmental stressors increases germline apoptosis. Our main results are (1) that blocking germ cell apoptosis using *ced-3* and *ced-4* mutants strongly reduces embryonic survival in stressful conditions that are likely to occur in the natural *C*. *elegans* habitat and (2) that these same genes are essential to maintain large, well-formed oocytes in these conditions.

Our study builds on work by Andux and Ellis [11]. In their study, they used mutants of the apoptosis genes, *ced-3* and *ced-4*, to show that maternal germline apoptosis is important for maintaining embryonic survival through preserving oocyte quality during aging. Oocytes produced by aging hermaphrodites are generally smaller and embryos produced from them exhibit higher embryonic lethality [11, 15]. Here we show that similar phenomena occur during *early* reproduction after exposure to different environmental stress conditions. We also show that apoptosis plays a protective role under these conditions, as it does during aging, preventing reductions in embryo survival, production, and size.

One explanation for the similarities between the effects of aging and exposure to these stresses, in terms of oocyte quality, may be that both reflect an effort on the part of the organism (through the apoptosis machinery) to produce quality gametes in the face of energy stress. Obviously, food deprivation can result in energy stress, but so does aging [38–40], as well as exposure to ethanol, oxidative stress, and acidic conditions [38, 41–49]. It is well-established that (though not well-understood how) the organism as a whole actively balances energy resources between growth/reproduction and maintenance of the soma [38, 50–52]. The declines we and others see in oocyte quality under physiological stress in the absence of *ced-3* and *ced-4* function speak to a role for the core apoptotic machinery in promoting this balance. We find at least three potential ways in which this pathway does so. We show that *ced-3* and *ced-4* are essential to maintain embryonic survival under stress and that this coincides with (1) decreases in embryo volume and (2) changes in embryo shape. We also show that there is (3) an overall reduction in the rate of embryo production in *ced-3* mutants and that this decreases further under stress. Each of these observations may reflect different functions of the core apoptotic machinery.

In the case of overall embryo production, it has been shown previously that *lf* mutations in *ced-3* and *ced-4* both result in smaller broods [11, 53]. This effect increases with age and in the presence of mutations that increase mtROS-mediated longevity and stress resistance while slowing growth and development [53–55]. Yee et al. [53] demonstrated that mtROS signals act through the intrinsic apoptosis pathway, without inducing apoptosis, to modulate gene expression in favor of slower growth and greater stress resistance [53]. We conclude that, while the effect of *ced-3* and *ced-4* mutations on brood size could be caused by apoptosis, it could also be the result of a signaling event involving the core apoptotic machinery and not apoptosis *per se*. Similarly, the role of the core apoptotic machinery in maintaining embryo survival under stress may be due to either apoptotic or ‘non-apoptotic’ roles of these proteins, though there is some controversy over the definition of ‘non-apoptotic’ functions of the caspase cascade (discussed in [16]).

Most ‘non-apoptotic’ roles of CED-3 in *C*. *elegans* (ex. mitochondrial dynamics, neuronal regeneration, stress resistance, embryonic development) have been demonstrated for development or maintenance of the soma [53, 56–61]. Of these, some are dependent on EGL-1 and/or inhibition of CED-9 [53, 56, 61], and others are not seen in *ced-3* single mutants [59, 60]. In this work, we demonstrated that the detrimental effect of *ced-3* and *ced-4 lf* mutations on embryonic survival were not recapitulated by *egl-1(lf)* or *ced-9(gf)*. It remains possible that the CED-3 stress-priming responses demonstrated by Weaver et al. may be involved in the phenotypes we see, although it remains unclear whether this stress-priming is dependent on EGL-1 [58]. It is also possible that there is either an uncharacterized non-apoptotic role of the caspase cascade affecting oocyte viability, or that the reduced embryonic survival is a direct effect of the absence of physiological germ cell apoptosis. Since the phenotypes we observe are dependent on the executioner caspase, CED-3, teasing apart the relative dependence on non-apoptotic cleavage events versus apoptosis is not trivial.

In their study on the role of apoptosis on aging oocytes [11], Andux and Ellis found that reduced embryonic survival was associated with reduced egg size in old in *ced-*3 and *ced-4* mothers, suggesting that physiological germ cell apoptosis was critical for adequate oocyte provisioning. Here we witness a similar situation in young mothers exposed environmental stress. Our data shows that *ced-3* and *ced-4* mutants (but not wildtype or *ced-9* mutants) respond to environmental stress by consistently producing embryos (oocytes) of significantly smaller, and more variable, size (Fig 6G). These results are thus most obviously explained by perturbed oocyte provisioning caused by a lack of stress-induced germ cell apoptosis. Recently, Chartier and colleagues have discovered a physical mechanism of hydraulic instability that determines which germ cells undergo apoptosis and might help explain how inhibiting apoptosis would result in insufficiently provisioned oocytes [13]. We also found that embryos of *ced-3* and *ced-4* mutants under stress exhibited strongly altered Width:Length ratios (shapes) (Fig 7A and 7B). Furthermore, embryo shape appears to a better predictor of embryonic survival than embryo volume in *ced-3(n718)* mutants (Fig 7C–7F). Observed spherical embryos with high mortality (Fig 7F) could potentially result from diverse perturbations during oocyte development, including incorrect provisioning or incomplete formation of the egg shell layers. However, how observed changes in embryo shape versus size relate to increased embryonic death remains to be tested more specifically, both in apoptosis-defective mutants and across multiple environmental conditions.

Taken together, we show here that physiological germ cell apoptosis is essential to preserve oocyte quality in young hermaphrodites exposed to various stressful environments. This phenomenon resembles that seen in aging mothers [11], and suggests that the apoptosis pathway plays a key role in maintaining reproduction when resources and energy become limited.

## Materials and methods

### Strains and maintenance

Nematodes were handled using standard methods [62–64]. All strains were maintained at 20°C on NGM agar plates seeded with the *E*.*coli* strain OP50. All strains were derived from the wild-type Bristol strain N2. The mutant alleles used in this study were: *ced-9(n1950gf)* (strain MT4770), *egl-1(n1084n3082)* (strain MT8735), *ced-3(n718)* (strain MT1743), *ced-3(n1286)* (MT3002), *ced-3(n2921)* (strain MT8309), *ced-4(n1162)* (strain MT2547), *ced-3(n718); fog-2(q71)* (strain RE415), *bcIs39 [P(lim-7)ced-1*::*GFP + lin-15(+)]* (strain MD701). Strains were obtained from Ron Ellis and the *Caenorhabditis* Genetics Center, which is funded by NIH Office of Research Infrastructure Programs (P40 OD010440).

### Environmental conditions

#### Starvation treatment

Liquid starvation was achieved by incubating animals in 1mL of S-basal buffer in a 24-well plate. The 24-well plate was kept under agitation for 20h at 20°C.

#### Paraquat

NGM plate agar was supplemented with paraquat (Methyl viologen dichloride hydrate; Sigma-Aldrich). A 1M stock solution was prepared, filtered and added to the autoclaved NGM medium for a final concentration of 0.5mM.

#### HCl

Hydrochloric acid (HCl; Sigma-Aldrich) was added to the NGM medium after autoclaving to reach a final concentration of 0.01M.

#### Ethanol

Chronic exposure to ethanol induces a delay in *C*. *elegans* development and a reduction in fecundity and lifespan [65]. Ethanol plates were made according to Davis et al. [65]. Briefly, ultrapure ethanol (VWR) was evenly pipetted onto OP50-seeded NGM plates in order to reach the desired ethanol concentration in the agar of 0.32M. The plates were then sealed with Parafilm and left to equilibrate for at least two hours at room temperature before use.

### Quantification of apoptosis

To assess the effects of diverse, ecologically relevant environmental stressors on germline apoptosis, we exposed young adult hermaphrodites bearing *ced-1*::GFP (MD701) [66] to 20h liquid starvation or NGM plates supplemented with HCl (0.01M, 12h), or paraquat (0.5mM, 12h). We also exposed worms from the L1 larval stage to 0.32M ethanol-supplemented plates until L4+24h [65]. Apoptotic corpses were quantified using the *P(lim-7)ced-1*::*GFP strain* (MD701), either in live animals under GFP fluorescence microscopy or after fixation of extruded gonads at L4+24h [66]. Live animals were imaged after being anesthetized with sodium azide and mounted in M9 medium on an agar pad. The GFP-positive cells were counted under epifluorescence. Images were taken at 40x magnification using an Olympus BX61 microscope with a CoolSnap HQ2 (Photometrics) camera.

### Effect of acid and oxidative stress on embryo production and survival

Populations of hermaphrodites were synchronized using hypochlorite treatment followed by a 27h L1-arrest in liquid without food. L1s were then transferred to control NGM plates seeded with OP50. At L4+12hr, hermaphrodites were transferred to new control, HCl-supplemented, or paraquat-supplemented NGM plates seeded with OP50 (see *Environmental Conditions* above). At L4+24h, single hermaphrodites from all conditions were transferred daily on control, seeded NGM plates and hatched larvae and dead embryos were counted. Eggs that did not hatch by 48h were scored as dead. The animals that died or disappeared during the reproductive period were not included in the analysis.

### Effect of ethanol on embryo production and survival

Populations of hermaphrodites were synchronized using hypochlorite treatment followed by a 27h L1-arrest in liquid without food. L1s were then transferred to ethanol-supplemented NGM plates (see *Environmental conditions* above). At L4+24h, all hermaphrodites were individualized on new control plates and transferred daily. Newly hatched larvae and dead embryos were counted. Importantly, Andux and Ellis [11] demonstrated a significant negative effect of *ced-3(lf)* on larval survival. Thus, we counted larvae during the L1 stage to ensure that egg viability was accurately scored. Eggs that did not hatch by 48h were scored as dead. The mothers that died or disappeared during the reproductive period were not included in the analysis.

Mating experiments were performed with *fog-2* and *fog-2;ced-3(n718)* mutants to test the role of apoptosis in the oogenic germline on embryo survival. Populations of *fog-2* and *fog-2;ced-3* females were synchronized and transferred from L1 to ethanol-supplemented NGM plates (see *Environmental conditions* above). L4+24h females were then put back on control plates and crossed with untreated males of the appropriate genotype. After 20h, single mated females were transferred daily to fresh control NGM plates and hatched larvae and dead embryos were counted. Eggs that did not hatch by 48h were scored as dead. The mothers that died or disappeared during the reproductive period were not included in the analysis.

### Effect of starvation on embryo production and survival

Populations of hermaphrodites were synchronized using hypochlorite treatment followed by a 27h L1-arrest in liquid without food. L1s were then transferred to control NGM plates seeded with OP50. At L4+20h, hermaphrodites were transferred to new control plates or subjected to liquid starvation stress (see *Environmental conditions* above). After 20h, single hermaphrodites were transferred daily on seeded NGM plates and newly hatched larvae and dead embryos were counted. Eggs that did not hatch by 48h were scored as dead. The mothers that died or disappeared during the reproductive period were not included in the analysis.

Mating experiments were performed with *fog-2* and *fog-2;ced-3* mutants to test the role of apoptosis in the oogenic germline on embryo survival during starvation. Populations of *fog-2* and *fog-2;ced-3* females were synchronized and grown on control OP50 plates. Young adult *fog-2* females (L4+20h) were washed 3 times and transferred in S-basal for 20h under agitation (see *Environment conditions* above). They were then crossed with untreated males of the appropriate genotype (20 females + 40 males per plate). After 20h, single mated females were transferred daily to fresh control NGM plates and hatched larvae and dead embryos were counted. Eggs that did not hatch by 48h were scored as dead. The mothers that died or disappeared during the reproductive period were not included in the analysis.

### Evaluation of embryo and oocyte size

After treatments, images of the embryos from multiple mothers were taken randomly from the plates using a binocular microscope at 10x. Embryo volume was calculated considering the oocytes as a prolate ellipsoid using the formula:

$$\frac{4}{3}\pi \left(\frac{length}{2}\right){\left(\frac{width}{2}\right)}^{2}$$

To image the oocytes, young adults (L4+24h) were mounted on 4% agar pads and observed under Differential Interference Contrast (DIC) microscopy at 40x magnification. Images were taken from an Olympus BX61 microscope with a CoolSnap HQ2 (Photometrics) camera.

### Statistical analysis and presentation

Generalized linear models were fitted to the data using the ‘glm’ or ‘lm” functions from the {stats} package in R (version 4.0.5, R.app GUI 1.74 (7950), S. Urbanek & H.-J. Bibiko, © R Foundation for Statistical Computing, 2020 [67]; RStudio 1.2.1335, © 2009–2018 RStudio, Inc.) and the {glmmTMB} R package version 1.0.2.1 [68]. Model evaluation was conducted with the aid of the {DHARMa} package version 0.4.1 according to the author’s guidelines [69]. Estimated marginal means and post-hoc pairwise comparisons (Tukey-adjusted) were calculated using the {emmeans} R package version 1.6.0 [70]. Charts were generated using the {ggplot2} R package version 3.3.3 [71] and the {ggbeeswarm} R package version 0.6.0 [72]. For all analyses, influential data points were defined as those having a Cook’s distance of 6/n and were excluded from the models and graphs. See S1–S10 Tables for details.

## Supporting information

S1 Data Complete raw data. (XLSX)

S1 Fig Apoptosis contributes to the maintenance of offspring production in stressful conditions. (A) Total number of embryos produced (living or dead) from L4+24h to L4+72h in wildtype (N2) and *ced-3(n718)* animals grown on control NGM plates, ethanol supplemented plates, or subjected to liquid starvation for 20h from L4+20h. (B) Total number of embryos produced (living or dead) from L4+24h to L4+72h in wildtype (N2) and *ced-3(n718)* animals exposed to HCl or paraquat supplemented plates or control NGM plates for 12 hours at L4+12h. Bold blue or red bars indicate estimated marginal means +/- standard error. (* p = 0.01–0.05, ** p = 0.001–0.01, *** p < 0.001; black asterisks indicate comparisons within environment while colored asterisks indicate comparisons within genotype). For statistical models (negative binomial regression) and results, see S10 Table.
(DOCX)

S1 Table (Accompanies Fig 1). Statistical testing for differences in the number of apoptotic corpses in wild type (ced-1::GFP) after acid or ethanol exposure, oxidative stress, or starvation.
(DOCX)

S2 Table (Accompanies Fig 2). Statistical testing for differences in embryonic survival in wild type (N2) versus apoptotic defective (ced-3) mutants after acid or ethanol exposure, oxidative stress, or starvation.
(DOCX)

S3 Table (Accompanies Fig 3). Statistical testing for differences in embryonic survival in wt (fog-2) versus apoptotic defective (fog-2;ced-3) mutants after ethanol exposure or starvation and mating to either wild type (fog-2) or apoptotic defective (fog-2;ced-3) males.
(DOCX)

S4 Table (Accompanies Fig 4). Statistical testing for differences in egg viability in wild type (N2) versus apoptotic defective (ced-3 and ced-4) mutants after ethanol exposure or starvation.
(DOCX)

S5 Table (Accompanies Fig 5). Statistical testing for differences in egg viability in wild type (N2) versus ced-3(n718), ced-9(gf), and egl-1(lf) mutants after ethanol exposure or starvation.
(DOCX)

S6 Table (Accompanies Fig 6). Statistical testing for differences in egg volume in wild type (N2), ced-9(n1950gf), and germline apoptosis defective (ced-3 and ced-4) mutants after ethanol exposure or starvation.
(DOCX)

S7 Table (Accompanies Fig 7A). Statistical testing for differences in egg shape (width:length) in wild type (N2), ced-9(n1950gf), and germline apoptosis defective (ced-3 and ced-4) mutants after ethanol exposure or starvation.
(DOCX)

S8 Table (Accompanies Fig 7B). Statistical testing for correlations between egg shape (width:length) and egg volume in wild type (N2), ced-9(n1950gf), and germline apoptosis defective (ced-3 and ced-4) mutants after ethanol exposure or starvation.
(DOCX)

S9 Table (Accompanies Fig 7C–7F). **Statistical testing for relationships between egg shape (width:length), egg length, egg width, or egg volume and hatching in ced-3(n718) mutants after starvation treatment.** Data were dummy coded and fitted to binomial models as shown below with logistic transformation using the glm function in R. For data representation, see Fig 7C–7F.
(DOCX)

S10 Table (Accompanies S1 Fig). Statistical testing for differences in embryo production in wild type (N2) versus apoptotic defective (ced-3) mutants after acid or ethanol exposure, oxidative stress, or starvation.
(DOCX)
