# Supplementary material for: Germ cell apoptosis is critical to maintain Caenorhabditis elegans offspring viability in stressful environments
Source: PLoS One. 2021 Dec 8;16(12):e0260573. doi: 10.1371/journal.pone.0260573 (PMC8654231; doi:10.1371/journal.pone.0260573)
Supplement: S1 Fig — (A) Total number of embryos produced (living or dead) from L4+24h to L4+72h in wildtype (N2) and ced-3(n718) animals grown on control NGM plates, ethanol supplemented plates, or subjected to liquid starvation for 20h from L4+20h. (B) Total number of embryos produced (living or dead) from L4+24h to L4+72h in wildtype (N2) and ced-3(n718) animals exposed to HCl or paraquat supplemented plates or control NGM plates for 12 hours at L4+12h. Bold blue or red bars indicate estimated marginal means +/- standard error. (* p = 0.01–0.05, ** p = 0.001–0.01, *** p < 0.001; black asterisks indicate comparisons within environment while colored asterisks indicate comparisons within genotype). For statistical models (negative binomial regression) and results, see S10 Table. (DOCX) [file pone.0260573.s002.docx]

**S1 Figure. Apoptosis contributes to the maintenance of offspring production in stressful conditions.** (A) Total number of embryos produced (living or dead) from L4+24h to L4+72h in wildtype (N2) and *ced-3(n718)* animals grown on control NGM plates, ethanol supplemented plates, or subjected to liquid starvation for 20h from L4+20h. (B) Total number of embryos produced (living or dead) from L4+24h to L4+72h in wildtype (N2) and *ced-3(n718)* animals exposed to HCl or paraquat supplemented plates or control NGM plates for 12 hours at L4+12h. Bold blue or red bars indicate estimated marginal means +/- standard error. (* p = 0.01-0.05, ** p = 0.001-0.01, *** p < 0.001; black asterisks indicate comparisons within environment while colored asterisks indicate comparisons within genotype). For statistical models (negative binomial regression) and results, see S10 Tables.
