# Supplementary material for: Germ cell apoptosis is critical to maintain Caenorhabditis elegans offspring viability in stressful environments
Source: PLoS One. 2021 Dec 8;16(12):e0260573. doi: 10.1371/journal.pone.0260573 (PMC8654231; doi:10.1371/journal.pone.0260573)
Supplement: S1 Table — Statistical testing for differences in the number of apoptotic corpses in wild type (ced-1::GFP) after acid or ethanol exposure, oxidative stress, or starvation. (DOCX) [file pone.0260573.s003.docx]

S1 Tables (accompanies Figure 1). Statistical testing for differences in the number of apoptotic corpses in wild type (*ced-1::GFP*) after acid or ethanol exposure, oxidative stress, or starvation. Data were fitted to negative binomial models (Corpses ~ Environment) with log transformation and overdispersion parameters of 1.1, 0.36, and 0.4, respectively (A, D, G). The R software package, ‘emmeans’ was used to obtain estimated marginal means on the response scale (B, E, H) and contrasts (C, F, I) with Tukey corrected p-values. For data representation, see Fig 1.

Table A. Germline apoptotic corpses after acid or oxidative stress: Conditional model

| Source | Estimate | SE | Z-value | Pr(>\|z\|) |  |
| --- | --- | --- | --- | --- | --- |
| Intercept | 1.7638 | 0.115 | 15.31 | <2e-16 | *** |
| EnvHCl | 0.3108 | 0.15 | 2.075 | 3.80E-01 | * |
| Envparaquat | 0.5472 | 0.142 | 3.854 | 0.000116 | *** |

**Table B. Germline apoptotic corpses after acid or oxidative stress: Emmeans**

| Environment | response | SE | df |
| --- | --- | --- | --- |
| control | 5.8348492 | 0.672140787 | 53 |
| HCl | 7.96135424 | 0.764826291 | 53 |
| paraquat | 10.0853509 | 0.839681776 | 53 |

**Table C. Germline apoptotic corpses after acid or oxidative stress: Contrasts**

| Contrast | ratio | SE | df | t-ratio | p -value |  |
| --- | --- | --- | --- | --- | --- | --- |
| control / HCl | 0.73289657 | 0.10976646 | 53 | -2.0748425 | 0.10484843 |  |
| control / paraquat | 0.57854697 | 0.08214305 | 53 | -3.8542699 | 0.00090787 | *** |

Table D. Germline apoptotic corpses after starvation: Conditional model

| Source | Estimate | SE | Z-value | Pr(>\|z\|) |  |
| --- | --- | --- | --- | --- | --- |
| Intercept | 0.192 | 0.211 | 0.909 | 3.63E-01 |  |
| Envstarved | 1.4808 | 0.235 | 6.308 | 2.84E-10 | *** |

**Table E. Germline apoptotic corpses after starvation: Emmeans**

| Env | response | SE | df |
| --- | --- | --- | --- |
| control | 1.21165847 | 0.25580229 | 49 |
| starved | 5.32679975 | 0.52957871 | 49 |

**Table F. Germline apoptotic corpses after starvation: Contrasts**

| Contrast | ratio | SE | df | t-ratio | p -value |  |
| --- | --- | --- | --- | --- | --- | --- |
| control/starved | 0.22746462 | 0.05339992 | 49 | -6.3075126 | 7.84E-08 | *** |

Table G. Germline apoptotic corpses after ethanol: Conditional model

| Source | Estimate | SE | Z-value | Pr(>\|z\|) |  |
| --- | --- | --- | --- | --- | --- |
| Intercept | 1.2043 | 0.156 | 7.733 | 1.05E-14 | *** |
| EnvEtOH | 1.4251 | 0.173 | 8.254 | <2e-16 | *** |

**Table H. Germline apoptotic corpses after ethanol: Emmeans**

| Env | response | SE | df |
| --- | --- | --- | --- |
| control | 3.33448333 | 0.51931193 | 47 |
| EtOH | 13.8655163 | 1.07426561 | 47 |

**Table I. Germline apoptotic corpses after ethanol: Contrasts**

| Contrast | ratio | SE | df | t-ratio | p -value |  |
| --- | --- | --- | --- | --- | --- | --- |
| control/EtOH | 0.2404875 | 0.04152198 | 47 | -8.2538363 | 1.07E-10 | *** |
