# Supplementary material for: Germ cell apoptosis is critical to maintain Caenorhabditis elegans offspring viability in stressful environments
Source: PLoS One. 2021 Dec 8;16(12):e0260573. doi: 10.1371/journal.pone.0260573 (PMC8654231; doi:10.1371/journal.pone.0260573)
Supplement: S2 Table — Statistical testing for differences in embryonic survival in wild type (N2) versus apoptotic defective (ced-3) mutants after acid or ethanol exposure, oxidative stress, or starvation. (DOCX) [file pone.0260573.s004.docx]

S2 Tables (accompanies Figure 2). Statistical testing for differences in embryonic survival in wild type (N2) versus apoptotic defective (*ced-*3) mutants after acid or ethanol exposure, oxidative stress, or starvation.

Data were fitted to beta binomial models (Survival rate ~ Genotype + Environment) weighted by the total number of embryos laid with logistic transformation and overdispersion parameters of 168 and 50.5, respectively (A, D). The R software package ‘Dharma’ was used to evaluate the models. Model (A) performed better when the interaction term was included, while model (D) did not. The R software package, ‘emmeans’ was used to obtain estimated marginal means on the response scale (B, E) and contrasts (C, F) with Tukey corrected p-values. For data representation, see Fig 2.

Table A. Embryonic survival rate after acid or oxidative stress: Conditional model

| Source | Estimate | SE | Z-value | Pr(>\|z\|) |  |
| --- | --- | --- | --- | --- | --- |
| Intercept | 5.6833 | 0.3804 | 14.942 | <2E-16 | *** |
| Geno ced-3 | -1.5177 | 0.4253 | -3.568 | 0.000359 | *** |
| Env HCl | -1.8196 | 0.4066 | -4.475 | 7.67E-06 | *** |
| Env paraquat | -1.6915 | 0.4216 | -4.012 | 6.02E-05 | *** |
| ced-3:HCl | -0.9019 | 0.4571 | -1.973 | 0.048506 | * |
| ced-3:paraquat | -0.4068 | 0.4725 | -0.861 | 0.389233 |  |

**Table B. Embryonic survival rate after** **acid or oxidative stress: Emmeans**

| Genotype | Environment | response | SE | df |
| --- | --- | --- | --- | --- |
| N2 | *control* | 0.996609143 | 0.001285343 | 95 |
| N2 | *HCl* | 0.979440197 | 0.003280829 | 95 |
| N2 | *paraquat* | 0.981868728 | 0.003447368 | 95 |
| ced-3 | *control* | 0.984716578 | 0.003163306 | 95 |
| ced-3 | *HCl* | 0.809085098 | 0.01045143 | 95 |
| ced-3 | *paraquat* | 0.887685055 | 0.008590123 | 95 |

**Table C. Embryonic survival rate after** **acid or oxidative stress: Contrasts**

| Env1 | Geno1 | Env2 | Geno2 | ratio | SE | df | t-ratio | p-value |  |
| --- | --- | --- | --- | --- | --- | --- | --- | --- | --- |
| control | *N2* | *HCl* | *N2* | 6.1696 | 2.5086 | 95 | 4.4752 | 2.99E-04 | *** |
| control | *N2* | *paraquat* | *N2* | 5.4274 | 2.2881 | 95 | 4.0122 | 1.63E-03 | ** |
| control | *ced-3* | *HCl* | *ced-3* | 15.2032 | 3.3431 | 95 | 11.0065 | 4.63E-10 | *** |
| control | *ced-3* | *paraquat* | *ced-3* | 8.1521 | 1.8551 | 95 | 9.2596 | 4.64E-10 | *** |
| control | *N2* | *control* | *ced-3* | 4.5617 | 1.9402 | 95 | 3.5684 | 7.26E-03 | ** |
| HCl | *N2* | *HCl* | *ced-3* | 11.2410 | 1.9735 | 95 | 13.7815 | 4.63E-10 | *** |
| paraquat | *N2* | *paraquat* | *ced-3* | 6.8518 | 1.4539 | 95 | 9.0699 | 4.64E-10 | *** |

Table D. Embryonic survival rate after ethanol or starvation: Conditional model

| Source | Estimate | SE | Z-value | Pr(>\|z\|) |  |
| --- | --- | --- | --- | --- | --- |
| Intercept | 6.8371 | 0.3793 | 18.027 | <2E-16 | *** |
| Geno ced-3 | -2.5165 | 0.2725 | -9.236 | <2E-16 | *** |
| Env EtOH | -2.446 | 0.2969 | -8.238 | <2E-16 | *** |
| Env starved | -1.5314 | 0.3185 | -4.808 | 1.52E-06 | *** |

**Table E. Embryonic survival rate after** **ethanol or starvation: Emmeans**

| Genotype | Environment | response | SE | df |
| --- | --- | --- | --- | --- |
| control | *N2* | 0.998927988 | 0.000406151 | 95 |
| EtOH | *N2* | 0.987764926 | 0.00322343 | 95 |
| starved | *N2* | 0.995061577 | 0.001483354 | 95 |
| control | *ced-3* | 0.986883427 | 0.003602774 | 95 |
| EtOH | *ced-3* | 0.866997524 | 0.014461317 | 95 |
| starved | *ced-3* | 0.942094271 | 0.009397765 | 95 |

**Table F. Embryonic survival rate after** **ethanol or starvation: Contrasts**

| Env1 | Geno1 | Env2 | Geno2 | ratio | SE | df | t-ratio | p-value |  |
| --- | --- | --- | --- | --- | --- | --- | --- | --- | --- |
| control | *N2* | *EtOH* | *N2* | 11.542 | 3.4270 | 95 | 8.2382 | 4.78E-10 | *** |
| control | *N2* | *starved* | *N2* | 4.6246 | 1.4729 | 95 | 4.8084 | 8.20E-05 | *** |
| control | *ced-3* | *EtOH* | *ced-3* | 11.542 | 3.4270 | 95 | 8.2382 | 4.78E-10 | *** |
| control | *ced-3* | *starved* | *ced-3* | 4.6246 | 1.4729 | 95 | 4.8084 | 8.20E-05 | *** |
| control | *N2* | *control* | *ced-3* | 12.384 | 3.3744 | 95 | 9.2360 | 4.64E-10 | *** |
| EtOH | *N2* | *EtOH* | *ced-3* | 12.384 | 3.3744 | 95 | 9.2360 | 4.64E-10 | *** |
| starved | *N2* | *starved* | *ced-3* | 12.384 | 3.3744 | 95 | 9.2360 | 4.64E-10 | *** |
