# Supplementary material for: Germ cell apoptosis is critical to maintain Caenorhabditis elegans offspring viability in stressful environments
Source: PLoS One. 2021 Dec 8;16(12):e0260573. doi: 10.1371/journal.pone.0260573 (PMC8654231; doi:10.1371/journal.pone.0260573)
Supplement: S3 Table — Statistical testing for differences in embryonic survival in wt (fog-2) versus apoptotic defective (fog-2;ced-3) mutants after ethanol exposure or starvation and mating to either wild type (fog-2) or apoptotic defective (fog-2;ced-3) males. (DOCX) [file pone.0260573.s005.docx]

S3 Tables (accompanies Figure 3). Statistical testing for differences in embryonic survival in wt (*fog-2*) versus apoptotic defective (*fog-2;ced-*3) mutants after ethanol exposure or starvation and mating to either wild type (*fog-2*) or apoptotic defective (*fog-2;ced-3*) males. Data were fitted to a beta binomial model (Survival rate ~ Female + Male + Environment + Female:Environment + Male:Environment) weighted by the total number of embryos laid with logistic transformation and an overdispersion parameter of 26.6 (A). The R software package ‘Dharma’ was used to evaluate the models. Model (A) performed better when the interaction terms Female:Environment and Male:Environment were included. The R software package, ‘emmeans’ was used to obtain estimated marginal means on the response scale (B) and contrasts (C-E) with Tukey corrected p-values. For data representation, see Fig 3.

Table A. Embryonic survival rate after ethanol or starvation: Conditional model

| Source | Estimate | SE | Z-value | Pr(>\|z\|) |  |
| --- | --- | --- | --- | --- | --- |
| Female ced-3 | 6.76727 | 0.61069 | 11.081 | <2E-16 | *** |
| Env EtOH | -3.37214 | 0.60019 | -5.618 | 1.93E-08 | *** |
| Env starved | -2.47088 | 0.71613 | -3.45 | 5.60E-04 | *** |
| Male ced-3 | -1.10376 | 0.69674 | -1.584 | 0.11315 |  |
| Female ced-3:Env EtOH | 0.04347 | 0.29604 | 0.147 | 0.88325 |  |
| Female ced-3:Env starved | 0.24347 | 0.70834 | 0.344 | 0.73106 |  |
| Env EtOH:Male ced-3 | -0.85449 | 0.7022 | -1.217 | 0.22365 |  |
| Env starved:Male ced-3 | -0.12047 | 0.35498 | -0.339 | 0.73433 |  |

**Table B. Embryonic survival rate after** **ethanol or starvation: Emmeans**

| Environment | Male | Female | response | SE | df |
| --- | --- | --- | --- | --- | --- |
| control | *wt* | *wt* | 0.9988505 | 0.00070118 | 204 |
| EtOH | *wt* | *wt* | 0.98656541 | 0.00522267 | 204 |
| starved | *wt* | *wt* | 0.9965417 | 0.0013034 | 204 |
| control | *ced-3* | *wt* | 0.99889935 | 0.00067015 | 204 |
| EtOH | *ced-3* | *wt* | 0.9855057 | 0.00528837 | 204 |
| starved | *ced-3* | *wt* | 0.99767094 | 0.00090443 | 204 |
| control | *wt* | *ced-3* | 0.96755219 | 0.00698165 | 204 |
| EtOH | *wt* | *ced-3* | 0.76273358 | 0.02471762 | 204 |
| starved | *wt* | *ced-3* | 0.80797267 | 0.018292 | 204 |
| control | *ced-3* | *ced-3* | 0.96888966 | 0.00689872 | 204 |
| EtOH | *ced-3* | *ced-3* | 0.74851872 | 0.02738418 | 204 |
| starved | *ced-3* | *ced-3* | 0.86215856 | 0.01779661 | 204 |

**Table C. Embryonic survival rate after** **ethanol or starvation: Contrasts-effect of maternal genotype**

| Env1 | **♂**1 | ♀1 | Env2 | **♂**2 | ♀2 | odds  ratio | SE | df | t-ratio | p-value |  |
| --- | --- | --- | --- | --- | --- | --- | --- | --- | --- | --- | --- |
| cntrl | *wt* | *wt* | cntrl | *wt* | *ced-3* | 29.1408 | 17.4900 | 204 | 5.6184 | 4.05E-06 | *** |
| cntrl | *ced-3* | *wt* | cntrl | *ced-3* | *ced-3* | 29.1408 | 17.4900 | 204 | 5.6184 | 4.05E-06 | *** |
| EtOH | *wt* | *wt* | *EtOH* | *wt* | *ced-3* | 22.8436 | 8.7376 | 204 | 8.1796 | 2.07E-12 | *** |
| EtOH | *ced-3* | *wt* | *EtOH* | *ced-3* | *ced-3* | 22.8436 | 8.7376 | 204 | 8.1796 | 2.07E-12 | *** |
| starve | *wt* | *wt* | *starve* | *wt* | *ced-3* | 68.4857 | 25.9348 | 204 | 11.161 | 1.53E-14 | *** |
| starve | *ced-3* | *wt* | *starve* | *ced-3* | *ced-3* | 68.4857 | 25.9348 | 204 | 11.161 | 1.53E-14 | *** |

**Table D. Embryonic survival rate after** **ethanol or starvation: Contrasts-effect of paternal genotype**

| Env1 | **♂**1 | ♀1 | Env2 | **♂**2 | ♀2 | odds  ratio | SE | df | t-ratio | p-value |  |
| --- | --- | --- | --- | --- | --- | --- | --- | --- | --- | --- | --- |
| cntrl | *wt* | *wt* | *cntrl* | *ced-3* | *wt* | 0.9575 | 0.2834 | 204 | -0.1469 | 1 |  |
| cntrl | *wt* | *ced-3* | *cntrl* | *ced-3* | *ced-3* | 0.9575 | 0.2834 | 204 | -0.1469 | 1 |  |
| EtOH | *wt* | *wt* | *EtOH* | *ced-3* | *wt* | 1.0800 | 0.2118 | 204 | 0.3927 | 1 |  |
| EtOH | *wt* | *ced-3* | *EtOH* | *ced-3* | *ced-3* | 1.0800 | 0.2118 | 204 | 0.3927 | 1 |  |
| starve | *wt* | *wt* | *starve* | *ced-3* | *wt* | 0.6727 | 0.1254 | 204 | -2.1263 | 0.605 |  |
| starve | *wt* | *ced-3* | *starve* | *ced-3* | *ced-3* | 0.6727 | 0.1254 | 204 | -2.1263 | 0.605 |  |

**Table E. Embryonic survival rate after** **ethanol or starvation: Contrasts-effects of environments**

| Env1 | **♂**1 | ♀1 | Env2 | **♂**2 | ♀2 | odds  ratio | SE | df | t-ratio | p-value |  |
| --- | --- | --- | --- | --- | --- | --- | --- | --- | --- | --- | --- |
| cntrl | *ced-3* | *wt* | *EtOH* | *ced-3* | *wt* | 13.3478 | 9.3970 | 204 | 3.6808 | 1.53E-02 | * |
| cntrl | *ced-3* | *wt* | *starve* | *ced-3* | *wt* | 2.1187 | 1.4908 | 204 | 1.0670 | 9.96E-01 |  |
| cntrl | *ced-3* | *ced-3* | *EtOH* | *ced-3* | *ced-3* | 10.4634 | 2.8182 | 204 | 8.7173 | 1.40E-13 | *** |
| cntrl | *ced-3* | *ced-3* | *starve* | *ced-3* | *ced-3* | 4.9792 | 1.3453 | 204 | 5.9413 | 7.85E-07 | *** |
| cntrl | *wt* | *wt* | *EtOH* | *wt* | *wt* | 11.8328 | 8.4738 | 204 | 3.4503 | 3.23E-02 | * |
| cntrl | *wt* | *wt* | *starve* | *wt* | *wt* | 3.0155 | 2.1010 | 204 | 1.5842 | 9.13E-01 |  |
| cntrl | *wt* | *ced-3* | *EtOH* | *wt* | *ced-3* | 9.2758 | 2.3824 | 204 | 8.6725 | 1.65E-13 | *** |
| cntrl | *wt* | *ced-3* | *starve* | *wt* | *ced-3* | 7.0869 | 1.7449 | 204 | 7.9532 | 8.17E-12 | *** |
