# Supplementary material for: Germ cell apoptosis is critical to maintain Caenorhabditis elegans offspring viability in stressful environments
Source: PLoS One. 2021 Dec 8;16(12):e0260573. doi: 10.1371/journal.pone.0260573 (PMC8654231; doi:10.1371/journal.pone.0260573)
Supplement: S4 Table — Statistical testing for differences in egg viability in wild type (N2) versus apoptotic defective (ced-3 and ced-4) mutants after ethanol exposure or starvation. (DOCX) [file pone.0260573.s006.docx]

**S4 Tables (accompanies Figure 4). Statistical testing for differences in egg viability in wild type (N2) versus apoptotic defective (*ced-*3 and *ced-4*) mutants after ethanol exposure or starvation.** Data were fitted to beta binomial models (Survival ~ Genotype * Environment) weighted by the total number of embryos laid with logistic transformation and a dispersion model (~Env) (A). The R software package ‘Dharma’ was used to evaluate the models. The R software package, ‘emmeans’ was used to obtain estimated marginal means on the response scale (B) and contrasts (C) with Tukey corrected p-values. For data representation, see Fig 4.

Table A. Proportion of surviving embryos after ethanol or starvation stress: Conditional model

| Source | Estimate | SE | Z-value | Pr(>\|z\|) |  |
| --- | --- | --- | --- | --- | --- |
| Intercept | 7.79446 | 1.0048 | 7.757 | 8.67e-15 | *** |
| Geno ced-3(n718) | -3.3656 | 1.0243 | -3.286 | 0.0010 | ** |
| Geno ced-3(n1286) | -2.0948 | 1.0589 | -1.978 | 0.0479 | * |
| Geno ced-3(n2921) | -3.1543 | 1.0291 | -3.065 | 0.0022 | ** |
| Geno ced-4(n1162) | 3.4789 | 1.0211 | -3.407 | 0.0007 | *** |
| Env EtOH | -2.0310 | 1.1640 | -1.745 | 0.0810 |  |
| Env Starvation | -3.1550 | 1.2368 | -2.551 | 0.0101 | * |
| Geno ced-3(n718):Env EtOH | 0.3170 | 1.1892 | 0.267 | 0.7898 |  |
| Geno ced-3(n1286):Env EtOH | -0.7423 | 1.2247 | -0.606 | 0.5445 |  |
| Geno ced-3(n2921):Env EtOH | -0.3143 | 1.1937 | -0.263 | 0.7923 |  |
| Geno ced-4(n1162):Env EtOH | 0.1862 | 1.1866 | 0.157 | 0.8753 |  |
| Geno ced-3(n718):Env Starvation | -0.0500 | 1.2719 | -0.039 | 0.9686 |  |
| Geno ced-3(n1286):Env Starvation | -1.1851 | 1.3004 | -0.909 | 0.3636 |  |
| Geno ced-3(n2921):Env Starvation | 0.2257 | 1.2849 | 0.176 | 0.8606 |  |
| Geno ced-4(n1162):Env Starvation | -0.0025 | 1.2733 | -0.002 | 0.9984 |  |
| Dispersion Source | *Estimate* | *SE* | *Z-value* | *Pr(>\|z\|)* |  |
| (Intercept) | 5.8780 | 0.6165 | 9.534 | <2e-16 | *** |
| Env EtOH | -2.1724 | 0.6752 | -3.217 | 0.0013 | ** |
| Env Starvation | -4.2590 | 0.6527 | -6.525 | 6.79e-11 | *** |

**Table B. Proportion of surviving embryos after ethanol or starvation stress: Emmeans**

| Genotype | Environment | prob | SE | df |
| --- | --- | --- | --- | --- |
| wt | *control* | 0.9996 | 0.0004 | 276 |
| ced-3(n718) | *control* | 0.9882 | 0.0024 | 276 |
| ced-3(n1286) | *control* | 0.9967 | 0.0011 | 276 |
| ced-3(n2921) | *control* | 0.9904 | 0.0022 | 276 |
| ced-4(n1162) | *control* | 0.9868 | 0.0026 | 276 |
| wt | *EtOH* | 0.9969 | 0.0018 | 276 |
| ced-3(n718) | *EtOH* | 0.9379 | 0.0098 | 276 |
| ced-3(n1286) | *EtOH* | 0.9491 | 0.0100 | 276 |
| ced-3(n2921) | *EtOH* | 0.9085 | 0.0135 | 276 |
| ced-4(n1162) | *EtOH* | 0.9221 | 0.0112 | 276 |
| wt | *Starvation* | 0.9904 | 0.0068 | 276 |
| ced-3(n718) | *Starvation* | 0.7727 | 0.0411 | 276 |
| ced-3(n1286) | *Starvation* | 0.7963 | 0.0389 | 276 |
| ced-3(n2921) | *Starvation* | 0.8469 | 0.0360 | 276 |
| ced-4(n1162) | *Starvation* | 0.7610 | 0.0455 | 276 |

**Table C. Proportion of surviving embryos after ethanol or starvation: Contrasts**

| Env1 | Geno1 | Env2 | Geno2 | ratio | SE | df | | t-ratio | | p-value |  | |  |
| --- | --- | --- | --- | --- | --- | --- | --- | --- | --- | --- | --- | --- | --- |
| control | *wt* | *control* | *ced-3(n718)* | 29.0 | 29.7 |  | 276 | | 0.075 | | |  | |
| control | *wt* | *control* | *ced-3(n1286)* | 8.12 | 8.60 |  | 276 | | 0.810 | | |  | |
| control | *wt* | *control* | *ced-3(n2921)* | 23.4 | 24.2 |  | 276 | | 0.136 | | |  | |
| control | *wt* | *control* | *ced-4(n1162)* | 32.4 | 33.1 |  | 276 | | 0.052 | | |  | |
| ethanol | *wt* | *ethanol* | *ced-3(n718)* | 21.1 | 12.7 |  | 276 | | 8.20E-05 | | | *** | |
| ethanol | *wt* | *ethanol* | *ced-3(n1286)* | 17.1 | 10.5 |  | 276 | | 0.0006 | | | *** | |
| ethanol | *wt* | *ethanol* | *ced-3(n2921)* | 32.1 | 19.4 |  | 276 | | 2.64E-06 | | | *** | |
| ethanol | *wt* | *ethanol* | *ced-4(n1162)* | 26.9 | 16.3 |  | 276 | | 1.16E-05 | | | *** | |
| starvation | *wt* | *starvation* | *ced-3(n718)* | 30. | 22.9 |  | 276 | | 0.0008 | | | *** | |
| starvation | *wt* | *starvation* | *ced-3(n1286)* | 26.5 | 20.0 |  | 276 | | 0.0018 | | | ** | |
| starvation | *wt* | *starvation* | *ced-3(n2921)* | 18.7 | 14.4 |  | 276 | | 0.0141 | | | * | |
| starvation | *wt* | *starvation* | *ced-4(n1162)* | 32.5 | 24.7 |  | 276 | | 0.0007 | | | *** | |
| control | *wt* | *ethanol* | *wt* | 7.62 | 8.87 |  | 276 | | 0.9170 | | |  | |
| control | *wt* | *starvation* | *wt* | 29.0 | 29.0 |  | 276 | | 0.4080 | | |  | |
| control | *ced-3(n718)* | *ethanol* | *ced-3(n718)* | 5.55 | 1.47 |  | 276 | | 4.87E-08 | | | *** | |
| control | *ced-3(n718)* | *starvation* | *ced-3(n718)* | 24.7 | 7.67 |  | 276 | | 6.95E-13 | | | *** | |
| control | *ced-3(n1286)* | *ethanol* | *ced-3(n1286)* | 16.0 | 6.4 |  | 276 | | 3.02E-09 | | | *** | |
| control | *ced-3(n1286)* | *starvation* | *ced-3(n1286)* | 76.4 | 32.0 |  | 276 | | 6.97E-13 | | | *** | |
| control | *ced-3(n2921)* | *ethanol* | *ced-3(n2921)* | 10.4 | 2.93 |  | 276 | | 1.08E-12 | | | *** | |
| control | *ced-3(n2921)* | *starvation* | *ced-3(n2921)* | 18.7 | 6.75 |  | 276 | | 2.30E-12 | | | *** | |
| control | *ced-4(n1162)* | *ethanol* | *ced-4(n1162)* | 6.32 | 1.59 |  | 276 | | 2.43E-12 | | | *** | |
| control | *ced-4(n1162)* | *starvation* | *ced-4(n1162)* | 23.5 | 7.48 |  | 276 | | 6.97E-13 | | | *** | |
