# Supplementary material for: Germ cell apoptosis is critical to maintain Caenorhabditis elegans offspring viability in stressful environments
Source: PLoS One. 2021 Dec 8;16(12):e0260573. doi: 10.1371/journal.pone.0260573 (PMC8654231; doi:10.1371/journal.pone.0260573)
Supplement: S5 Table — Statistical testing for differences in egg viability in wild type (N2) versus ced-3(n718), ced-9(gf), and egl-1(lf) mutants after ethanol exposure or starvation. (DOCX) [file pone.0260573.s007.docx]

**S5 Tables (accompanies Figure 5). Statistical testing for differences in egg viability in wild type (N2) versus *ced-3(n718)*, *ced-9(gf),* and *egl-1(lf)* mutants after ethanol exposure or starvation.** Data were fitted to beta binomial models (Survival ~ Genotype * Environment) weighted by the total number of embryos laid with logistic transformation and an overdispersion parameter of 103 (A). The R software package ‘Dharma’ was used to evaluate the models. The R software package, ‘emmeans’ was used to obtain estimated marginal means on the response scale (B) and contrasts (C) with Tukey corrected p-values. For data representation, see Fig 5.

Table A. Proportion of surviving embryos after ethanol or starvation stress: Conditional model

| Source | Estimate | SE | Z-value | Pr(>\|z\|) |  |
| --- | --- | --- | --- | --- | --- |
| Intercept | 7.6446 | 1.0059 | 7.600 | 2.97E-14 | *** |
| Geno ced-3(n718) | -3.3165 | 1.0310 | -3.217 | 0.0013 | ** |
| Geno ced-9(n1960gf) | -3.0821 | 1.0385 | -2.968 | 0.0030 | ** |
| Geno egl-1(n1084n3082) | -1.7275 | 1.1178 | -1.545 | 0.1222 |  |
| Env EtOH | -2.7015 | 1.0516 | -2.569 | 0.0102 | * |
| Env Starvation | -2.4230 | 1.0608 | -2.284 | 0.0224 | * |
| Geno ced-3(n718):Env EtOH | 1.0538 | 1.0867 | 0.970 | 0.3322 |  |
| Geno ced-9(n1960gf):Env EtOH | 2.6259 | 1.1282 | 2.328 | 0.0199 | * |
| Geno egl-1(n1084n3082):Env EtOH | 0.8578 | 1.2013 | 0.714 | 0.4752 |  |
| Geno ced-3(n718):Env Starvation | 0.3062 | 1.0940 | 0.280 | 0.7796 |  |
| Geno ced-9(n1960gf):Env Starvation | 1.9348 | 1.1205 | 1.727 | 0.0842 |  |
| Geno egl-1(n1084n3082):Env Starvation | 20.006 | 4728.1 | 0.004 | 0.9966 |  |

**Table B. Proportion of surviving embryos after ethanol or starvation stress: Emmeans**

| Genotype | Environment | prob | SE | df |
| --- | --- | --- | --- | --- |
| wt | *control* | 0.9995 | 0.0005 | 226 |
| ced-3(n718) | *control* | 0.9870 | 0.0031 | 226 |
| ced-9(n1950) | *control* | 0.9897 | 0.0028 | 226 |
| egl-1(n1084n3082) | *control* | 0.9973 | 0.0013 | 226 |
| wt | *EtOH* | 0.9929 | 0.0023 | 226 |
| ced-3(n718) | *EtOH* | 0.9359 | 0.0083 | 226 |
| ced-9(n1950) | *EtOH* | 0.9889 | 0.0034 | 226 |
| egl-1(n1084n3082) | *EtOH* | 0.9833 | 0.0050 | 226 |
| wt | *Starvation* | 0.9946 | 0.0019 | 226 |
| ced-3(n718) | *Starvation* | 0.9013 | 0.0106 | 226 |
| ced-9(n1950) | *Starvation* | 0.9833 | 0.0039 | 226 |
| egl-1(n1084n3082) | *Starvation* | 1.0000 | 2.94E-07 | 226 |

**Table C. Proportion of surviving embryos after ethanol or starvation: Contrasts**

| Env1 | Geno1 | Env2 | Geno2 | odds  ratio | SE | df | t-ratio | p-value |  |
| --- | --- | --- | --- | --- | --- | --- | --- | --- | --- |
| cntrl | *wt* | *cntrl* | *ced-3(n718)* | 27.6 | 28.4 | 226 | 3.22 | 0.064 |  |
| cntrl | *wt* | *cntrl* | *ced-9(n1950gf))* | 21.8 | 22.6 |  | 2.97 | 0.125 |  |
| cntrl | *wt* | *cntrl* | *egl-1(n1084n3082)* | 5.63 | 6.29 |  | 1.55 | 0.926 |  |
| EtOH | *wt* | *EtOH* | *ced-3(n718)* | 9.61 | 3.38 |  | 6.43 | 5.03E-08 | *** |
| EtOH | *wt* | *EtOH* | *ced-9(n1950gf))* | 1.58 | 0.697 |  | 1.03 | 0.997 |  |
| EtOH | *wt* | *EtOH* | *egl-1(n1084n3082)* | 2.39 | 1.06 |  | 1.96 | 0.717 |  |
| starve | *wt* | *starve* | *ced-3(n718)* | 20.3 | 7.71 |  | 7.92 | 2.43E-09 | *** |
| starve | *wt* | *starve* | *ced-9(n1950gf))* | 3.15 | 1.35 |  | 2.68 | 1 |  |
| starve | *wt* | *starve* | *egl-1(n1084n3082)* | 1.15E-08 | 5.5E-05 |  | -0.004 | 1 |  |
| cntrl | *wt* | *EtOH* | *wt* | 14.9 | 15.7 |  | 2.57 | 0.304 |  |
| cntrl | *wt* | *starve* | *wt* | 11.3 | 12.0 |  | 2.28 | 0.491 |  |
| cntrl | *ced-3(n718)* | *EtOH* | *ced-3(n718)* | 5.19 | 1.45 |  | 5.92 | 7.71E-07 | *** |
| cntrl | *ced-3(n718)* | *starve* | *ced-3(n718)* | 8.30 | 0.175 |  | -2.09 | 1.52E-11 | *** |
| cntrl | *ced-9(n1950gf))* | *EtOH* | *ced-9(n1950gf))* | 1.08 | 0.288 |  | 0.185 | 1 |  |
| cntrl | *ced-9(n1950gf))* | *starve* | *ced-9(n1950gf))* | 1.63 | 0.231 |  | -1.48 | 0.972 |  |
| cntrl | *egl-1(n1084n3082)* | *EtOH* | *egl-1(n1084n3082)* | 6.32 | 1.57 |  | 3.15 | 0.078 |  |
| cntrl | *egl-1(n1084n3082)* | *starve* | *egl-1(n1084n3082)* | 2.31E-08 | 1.22 |  | -0.004 | 1 |  |
