# Supplementary material for: Germ cell apoptosis is critical to maintain Caenorhabditis elegans offspring viability in stressful environments
Source: PLoS One. 2021 Dec 8;16(12):e0260573. doi: 10.1371/journal.pone.0260573 (PMC8654231; doi:10.1371/journal.pone.0260573)
Supplement: S6 Table — Statistical testing for differences in egg volume in wild type (N2), ced-9(n1950gf), and germline apoptosis defective (ced-3 and ced-4) mutants after ethanol exposure or starvation. (DOCX) [file pone.0260573.s008.docx]

S6 Tables (accompanies Figure 6). Statistical testing for differences in egg volume in wild type (N2), *ced-9(n1950gf)*, and germline apoptosis defective (*ced-3* and *ced-4*) mutants after ethanol exposure or starvation. Data were fitted to a gaussian model (Egg volume ~ Genotype* Environment) with no transformation including a dispersion formula (~Env). The R software package ‘Dharma’ was used to evaluate the model. The R software package, ‘emmeans’ was used to obtain means (B) and contrasts (C) with Tukey corrected p-values. For data representation, see Fig 6.

Table A. Egg volume after ethanol exposure or starvation: model

| Source | Estimate | SE | Z-value | Pr(>\|z\|) | |  | |  |
| --- | --- | --- | --- | --- | --- | --- | --- | --- |
| Intercept | 22825.94 | 355.40 | 64.23 | <2E-16 | | *** | |  |
| Geno ced-9(n1950) | -339.29 | 664.36 | -0.51 | 0.6096 | |  | |  |
| Geno ced-3(n718) | 1266.23 | 504.83 | 2.51 | 0.0121 | | * | |  |
| Geno ced-3(n1286) | 657.37 | 575.72 | 1.14 | 0.2535 | |  | |  |
| Geno ced-3(n2921) | 274.68 | 578.16 | 0.48 | 0.6347 | |  | |  |
| Geno ced-4(n1162) | 5822.89 | 619.26 | 9.40 | <2E-16 | | *** | |  |
| Env EtOH | 42.95 | 552.41 | 0.08 | 0.9380 | |  | |  |
| Env Starvation | -1533.2 | 635.43 | -2.41 | 0.0158 | | * | |  |
| Geno ced-9(n1950):Env EtOH | -2216.3 | 1009.54 | -2.20 | 0.0281 | | * | |  |
| Geno ced-3(n718):Env EtOH | -4097.39 | 763.78 | -5.36 | 8.11E-08 | | *** | |  |
| Geno ced-3(n1286):Env EtOH | -2687.76 | 909.84 | -2.95 | 0.0031 | | ** | |  |
| Geno ced-3(n2921):Env EtOH | -4171.33 | 799.12 | -5.22 | 1.79E-07 | | *** | |  |
| Geno ced-4(n1162):Env EtOH | -8102.84 | 938.00 | -8.64 | <2.E-16 | | *** | |  |
| Geno ced-9(n1950):Env Starvation | -91.63 | 1136.53 | -0.08 | 0.9357 | |  | |  |
| Geno ced-3(n718):Env Starvation | -4806.63 | 898.29 | -5.35 | 8.75E-08 | | *** | |  |
| Geno ced-3(n1286):Env Starvation | -6574.46 | 1002.33 | -6.56 | 5.41E-11 | | *** | |  |
| Geno ced-3(n2921):Env Starvation | -2486.30 | 895.86 | -2.78 | 0.0055 | | ** | |  |
| Geno ced-4(n1162):Env Starvation | -7880.03 | 1039.91 | -7.58 | 3.52E-14 | | *** | |  |
| Dispersion Source | *Estimate* | *SE* | *Z-value* | | *Pr(>\|z\|)* | |  | |
| (Intercept) | 16.465 | 0.0661 | 249.11 | | <2E-16 | | *** | |
| Env EtOH | 0.0954 | 0.0938 | 1.02 | | 0.309 | |  | |
| Env Starvation | 0.6433 | 0.0909 | 7.08 | | 1.43E-12 | | *** | |

**Table B. Egg volume after ethanol exposure or starvation: Emmeans**

| Genotype | Environment | emmean | SE | df |
| --- | --- | --- | --- | --- |
| wt | *control* | 22739.21 | 416.61 | 1408 |
| ced-9(n1950) | *control* | 22829.22 | 657.25 | 1408 |
| ced-3(n718) | *control* | 24014.83 | 420.38 | 1408 |
| ced-3(n1286) | *control* | 23611.81 | 530.78 | 1408 |
| ced-3(n2921) | *control* | 23043.34 | 534.67 | 1408 |
| ced-4(n1162) | *control* | 28680.47 | 594.51 | 1408 |
| wt | *EtOH* | 22822.91 | 472.69 | 1408 |
| ced-9(n1950) | *EtOH* | 20190.22 | 706.00 | 1408 |
| ced-3(n718) | *EtOH* | 20124.08 | 432.33 | 1408 |
| ced-3(n1286) | *EtOH* | 20739.45 | 629.85 | 1408 |
| ced-3(n2921) | *EtOH* | 18990.49 | 395.94 | 1408 |
| ced-4(n1162) | *EtOH* | 20583.46 | 629.85 | 1408 |
| wt | *Starvation* | 21366.30 | 447.66 | 1408 |
| ced-9(n1950) | *Starvation* | 20646.98 | 643.11 | 1408 |
| ced-3(n718) | *Starvation* | 17723.29 | 445.37 | 1408 |
| ced-3(n1286) | *Starvation* | 15354.13 | 534.67 | 1408 |
| ced-3(n2921) | *Starvation* | 19102.79 | 371.30 | 1408 |
| ced-4(n1162) | *Starvation* | 19261.63 | 551.12 | 1408 |

**Table C. Egg volume after ethanol exposure or starvation: Contrasts**

| Env1 | Geno1 | Env2 | Geno2 | est | SE | df | t-ratio | p-value |  |
| --- | --- | --- | --- | --- | --- | --- | --- | --- | --- |
| cntrl | *wt* | *cntrl* | *ced-9(n1950)* | -90.0 | 778.2 | 1408 | -0.116 | 1 |  |
| cntrl | *wt* | *cntrl* | *ced-3(n718)* | -1275.6 | 591.8 | 1408 | -2.155 | 0.7827 |  |
| cntrl | *wt* | *cntrl* | *ced-3(n1286)* | -872.59 | 674.7 | 1408 | -1.2932 | 0.9983 |  |
| cntrl | *wt* | *cntrl* | *ced-3(n2921)* | -304.12 | 677.8 | 1408 | -0.4487 | 1 |  |
| cntrl | *wt* | *cntrl* | *ced-4(n1162)* | -5941.3 | 725.9 | 1408 | -8.1842 | 0 | *** |
| EtOH | *wt* | *EtOH* | *ced-9(n1950)* | 2632.7 | 849.6 | 1408 | 3.099 | 0.158 |  |
| EtOH | *wt* | *EtOH* | *ced-3(n718)* | 2698.8 | 640.6 | 1408 | 4.213 | 0.004 | ** |
| EtOH | *wt* | *EtOH* | *ced-3(n1286)* | 2083.5 | 787.5 | 1408 | 2.646 | 0.420 |  |
| EtOH | *wt* | *EtOH* | *ced-3(n2921)* | 3832.4 | 616.6 | 1408 | 6.215 | 1.03E-7 | *** |
| EtOH | *wt* | *EtOH* | *ced-4(n1162)* | 2239.5 | 787.5 | 1408 | 2.844 | 0.2867 |  |
| starve | *wt* | *starve* | *ced-9(n1950)* | 719.32 | 783.6 | 1408 | 0.9180 | 1 |  |
| starve | *wt* | *starve* | *ced-3(n718)* | 3643.0 | 631.5 | 1408 | 5.7691 | 1.48E-6 | *** |
| starve | *wt* | *starve* | *ced-3(n1286)* | 6012.2 | 697.3 | 1408 | 8.6217 | 0 | *** |
| starve | *wt* | *starve* | *ced-3(n2921)* | 2263.5 | 581.6 | 1408 | 3.8918 | 0.0126 | * |
| starve | *wt* | *starve* | *ced-4(n1162)* | 2104.7 | 710.0 | 1408 | 2.9642 | 0.2192 |  |
| cntrl | *wt* | *EtOH* | *wt* | -83.695 | 630.1 | 1408 | -0.1328 | 1 |  |
| cntrl | *wt* | *starve* | *wt* | 1372.91 | 611.5 | 1408 | 2.2451 | 0.723 |  |
| cntrl | *ced-9(n1950)* | *EtOH* | *ced-9(n1950* | 2639.0 | 964.6 | 1408 | 2.7359 | 0.3563 |  |
| cntrl | *ced-9(n1950)* | *starve* | *ced-9(n1950* | 2182.2 | 919.6 | 1408 | 2.3732 | 0.6286 |  |
| cntrl | *ced-3(n718)* | *EtOH* | *ced-3(n718)* | 3890.7 | 603.0 | 1408 | 6.4521 | 2.31E-8 | *** |
| cntrl | *ced-3(n718)* | *starve* | *ced-3(n718)* | 6291.5 | 612.4 | 1408 | 10.273 | 0 | *** |
| cntrl | *ced-3(n1286)* | *EtOH* | *ced-3(n1286)* | 2872.4 | 823.7 | 1408 | 3.4872 | 0.05 | * |
| cntrl | *ced-3(n1286)* | *starve* | *ced-3(n1286)* | 8257.7 | 753.4 | 1408 | 10.961 | 0 | *** |
| cntrl | *ced-3(n2921)* | *EtOH* | *ced-3(n2921)* | 4052.8 | 665.3 | 1408 | 6.0917 | 2.19E-7 | *** |
| cntrl | *ced-3(n2921)* | *starve* | *ced-3(n2921)* | 3940.6 | 650.9 | 1408 | 6.0536 | 2.76E-7 | *** |
| cntrl | *ced-4(n1162)* | *EtOH* | *ced-4(n1162)* | 80.97.0 | 866.1 | 1408 | 9.3487 | 0 | *** |
| cntrl | *ced-4(n1162)* | *starve* | *ced-4(n1162)* | 9418.8 | 810.7 | 1408 | 11.619 | 0 | *** |
