# Supplementary material for: Germ cell apoptosis is critical to maintain Caenorhabditis elegans offspring viability in stressful environments
Source: PLoS One. 2021 Dec 8;16(12):e0260573. doi: 10.1371/journal.pone.0260573 (PMC8654231; doi:10.1371/journal.pone.0260573)
Supplement: S7 Table — Statistical testing for differences in egg shape (width:length) in wild type (N2), ced-9(n1950gf), and germline apoptosis defective (ced-3 and ced-4) mutants after ethanol exposure or starvation. (DOCX) [file pone.0260573.s009.docx]

S7 Tables (accompanies Figure 7A). Statistical testing for differences in egg shape (width:length) in wild type (N2), *ced-9(n1950gf)*, and germline apoptosis defective (*ced-3* and *ced-4*) mutants after ethanol exposure or starvation. Data were fitted to a gaussian model (WLratio ~ Genotype* Environment) with no transformation including a dispersion formula (~Environment + Genotype:Environment). The R software package ‘Dharma’ was used to evaluate the model. The R software package, ‘emmeans’ was used to obtain means (B) and contrasts (C) with Tukey corrected p-values. For data representation, see Fig 7A.

Table A. Egg shape (width:length) after ethanol exposure or starvation: model

| Source | Estimate | SE | Z-value | Pr(>\|z\|) | |  | |  |
| --- | --- | --- | --- | --- | --- | --- | --- | --- |
| Intercept | 0.5842 | 0.0039 | 148.84 | <2E-16 | | *** | |  |
| Geno ced-9(n1950) | 0.0070 | 0.0087 | 0.81 | 0.4189 | |  | |  |
| Geno ced-3(n718) | 0.0161 | 0.0062 | 2.61 | 0.0090 | | ** | |  |
| Geno ced-3(n1286) | 0.0458 | 0.0103 | 4.44 | 8.83E-06 | | *** | |  |
| Geno ced-3(n2921) | 0.0007 | 0.0070 | 0.10 | 0.9213 | |  | |  |
| Geno ced-4(n1162) | 0.0165 | 0.0083 | 1.98 | 0.0475 | | * | |  |
| Env EtOH | 0.0362 | 0.0065 | 5.59 | 2.32E-08 | | *** | |  |
| Env Starvation | 0.0019 | 0.0057 | 0.33 | 0.7417 | |  | |  |
| Geno ced-9(n1950):Env EtOH | 0.0161 | 0.0153 | 1.05 | 0.2925 | |  | |  |
| Geno ced-3(n718):Env EtOH | 0.0088 | 0.0100 | 0.88 | 0.3785 | |  | |  |
| Geno ced-3(n1286):Env EtOH | -0.0523 | 0.0138 | -3.78 | 0.0002 | | *** | |  |
| Geno ced-3(n2921):Env EtOH | 0.0766 | 0.0137 | 5.61 | 1.98E-08 | | *** | |  |
| Geno ced-4(n1162):Env EtOH | -0.0138 | 0.01332 | -1.04 | 0.2997 | |  | |  |
| Geno ced-9(n1950):Env Starvation | 0.0137 | 0.0119 | 1.15 | 0.2507 | |  | |  |
| Geno ced-3(n718):Env Starvation | 0.0964 | 0.0134 | 7.18 | 6.78E-13 | | *** | |  |
| Geno ced-3(n1286):Env Starvation | 0.1000 | 0.0218 | 4.59 | 4.35E-06 | | *** | |  |
| Geno ced-3(n2921):Env Starvation | 0.0847 | 0.0127 | 6.69 | 2.27E-11 | | *** | |  |
| Geno ced-4(n1162):Env Starvation | 0.0627 | 0.0169 | 3.72 | 0.0002 | | *** | |  |
| Dispersion Source | *Estimate* | *SE* | *Z-value* | | *Pr(>\|z\|)* | |  | |
| (Intercept) | -63621 | 0.1336 | -47.61 | | <2E-16 | | *** | |
| Env EtOH | 0.2937 | 0.2021 | 1.45 | | 0.1462 | |  | |
| Env Starvation | -0.0352 | 0.1962 | -0.18 | | 0.8577 | |  | |
| Env Control:Geno ced-9(n1950) | 0.4415 | 0.2496 | 1.77 | | 0.0769 | |  | |
| Env EtOH:Geno ced-9(n1950) | 0.8113 | 0.2725 | 2.98 | | 0.0029 | | ** | |
| Env Starvation:Geno ced-9(n1950) | 0.3394 | 0.2513 | 1.35 | | 0.1769 | |  | |
| Env Control:Geno ced-3(n718) | 0.3722 | 0.1898 | 1.96 | | 0.0499 | | * | |
| Env EtOH:Geno ced-3(n718) | 0.4379 | 0.2055 | 2.13 | | 0.0331 | | * | |
| Env Starvation:Geno ced-3(n718) | 1.9930 | 0.2026 | 9.84 | | <2E-16 | | *** | |
| Env Control:Geno ced-3(n1286) | 1.2915 | 0.2164 | 5.97 | | 2.41E-09 | | *** | |
| Env EtOH:Geno ced-3(n1286) | 0.2117 | 0.2526 | 0.84 | | 0.4860 | |  | |
| Env Starvation:Geno ced-3(n1286) | 2.6606 | 0.2237 | 11.89 | | <2E-16 | | *** | |
| Env Control:Geno ced-3(n2921) | 0.1515 | 0.2174 | 0.70 | | 0.4860 | |  | |
| Env EtOH:Geno ced-3(n2921) | 1.8163 | 0.1978 | 9.18 | | <2E-16 | | *** | |
| Env Starvation:Geno ced-3(n2921) | 2.1204 | 0.1866 | 11.37 | | <2E-16 | | *** | |
| Env Control:Geno ced-4(n1162) | 0.5381 | 0.2329 | 2.31 | | 0.0208 | | * | |
| Env EtOH:Geno ced-4(n1162) | 0.5483 | 0.2526 | 2.17 | | 0.0300 | | * | |
| Env Starvation:Geno ced-4(n1162) | 2.0271 | 0.2278 | 8.90 | | <2E-16 | | *** | |

**Table B. Egg shape (width:length) after ethanol exposure or starvation: Emmeans**

| Genotype | Environment | emmean | SE | df |
| --- | --- | --- | --- | --- |
| wt | *control* | 0.5846 | 0.0039 | 1390 |
| ced-9(n1950) | *control* | 0.5913 | 0.0077 | 1390 |
| ced-3(n718) | *control* | 0.6004 | 0.0048 | 1390 |
| ced-3(n1286) | *control* | 0.6301 | 0.0095 | 1390 |
| ced-3(n2921) | *control* | 0.5849 | 0.0054 | 1390 |
| ced-4(n1162) | *control* | 0.6007 | 0.0073 | 1390 |
| wt | *EtOH* | 0.6205 | 0.0052 | 1390 |
| ced-9(n1950) | *EtOH* | 0.6436 | 0.0116 | 1390 |
| ced-3(n718) | *EtOH* | 0.6454 | 0.0059 | 1390 |
| ced-3(n1286) | *EtOH* | 0.6140 | 0.0076 | 1390 |
| ced-3(n2921) | *EtOH* | 0.6978 | 0.0107 | 1390 |
| ced-4(n1162) | *EtOH* | 0.6231 | 0.0090 | 1390 |
| wt | *Starvation* | 0.5861 | 0.0041 | 1390 |
| ced-9(n1950) | *Starvation* | 0.6068 | 0.0071 | 1390 |
| ced-3(n718) | *Starvation* | 0.6987 | 0.0112 | 1390 |
| ced-3(n1286) | *Starvation* | 0.7320 | 0.0187 | 1390 |
| ced-3(n2921) | *Starvation* | 0.6715 | 0.0099 | 1390 |
| ced-4(n1162) | *Starvation* | 0.6653 | 0.0141 | 1390 |

**Table C. Egg shape (width:length) after ethanol exposure or starvation: Contrasts**

| Env1 | Geno1 | Env2 | Geno2 | estimate | SE | df | t-ratio | p-value |  |
| --- | --- | --- | --- | --- | --- | --- | --- | --- | --- |
| cntrl | *wt* | *cntrl* | *ced-9(n1950)* | -0.0070 | 0.009 | 1390 | -0.8083 | 1 |  |
| cntrl | *wt* | *cntrl* | *ced-3(n718)* | -0.0161 | 0.006 |  | -2.6127 | 0.4446 |  |
| cntrl | *wt* | *cntrl* | *ced-3(n1286)* | -0.0458 | 0.010 |  | -4.4440 | 0.0013 | ** |
| cntrl | *wt* | *cntrl* | *ced-3(n2921)* | -0.0007 | 0.007 |  | 0.0988 | 1 |  |
| cntrl | *wt* | *cntrl* | *ced-4(n1162)* | -0.0165 | 0.008 |  | -1.9822 | 0.8776 |  |
| EtOH | *wt* | *EtOH* | *ced-9(n1950)* | -0.0231 | 0.013 |  | -1.8288 | 0.9357 |  |
| EtOH | *wt* | *EtOH* | *ced-3(n718)* | -0.0249 | 0.008 |  | -3.1877 | 0.1245 |  |
| EtOH | *wt* | *EtOH* | *ced-3(n1286)* | 0.0065 | 0,009 |  | 0.7032 | 1 |  |
| EtOH | *wt* | *EtOH* | *ced-3(n2921)* | -0.0773 | 0.012 |  | -6.5002 | 1.70E-8 | *** |
| EtOH | *wt* | *EtOH* | *ced-4(n1162)* | -0.0027 | 0.010 |  | -0.2562 | 1 |  |
| starve | *wt* | *starve* | *ced-9(n1950)* | -0.0207 | 0.008 |  | -2.5285 | 0.5086 |  |
| starve | *wt* | *starve* | *ced-3(n718)* | -0.1125 | 0.012 |  | -9.4474 | 0 | *** |
| starve | *wt* | *starve* | *ced-3(n1286)* | -0.1457 | 0.019 |  | -7.6071 | 7.2E-12 | *** |
| starve | *wt* | *starve* | *ced-3(n2921)* | -0.0854 | 0.010 |  | -7.9416 | 5E-14 | *** |
| starve | *wt* | *starve* | *ced-4(n1162)* | 0.0792 | 0.015 |  | -5.4028 | 1.15E-5 | *** |
| cntrl | *wt* | *EtOH* | *wt* | -0.0362 | 0.007 |  | -5.5863 | 4.20E-6 | *** |
| cntrl | *wt* | *starve* | *wt* | -0.0019 | 0.006 |  | -0.3296 | 1 |  |
| cntrl | *ced-9(n1950)* | *EtOH* | *ced-9(n1950* | -0.0524 | 0.014 |  | -3.7664 | 0.0200 | * |
| cntrl | *ced-9(n1950)* | *starve* | *ced-9(n1950* | -0.0156 | 0.010 |  | -1.4884 | 0.9914 |  |
| cntrl | *ced-3(n718)* | *EtOH* | *ced-3(n718)* | -0.0450 | 0.008 |  | -5.9452 | 5.29E-7 | *** |
| cntrl | *ced-3(n718)* | *starve* | *ced-3(n718)* | -0.0983 | 0.012 |  | -8.0926 | 0 | *** |
| cntrl | *ced-3(n1286)* | *EtOH* | *ced-3(n1286)* | 0.0161 | 0.012 |  | 1.3184 | 0.9979 |  |
| cntrl | *ced-3(n1286)* | *starve* | *ced-3(n1286)* | -0.1019 | 0.021 |  | -4.8499 | 0.0002 | *** |
| cntrl | *ced-3(n2921)* | *EtOH* | *ced-3(n2921)* | -0.1128 | 0.012 |  | -9.3931 | 0 | *** |
| cntrl | *ced-3(n2921)* | *starve* | *ced-3(n2921)* | -0.0866 | 0.011 |  | -7.6565 | 4.8E-12 | *** |
| cntrl | *ced-4(n1162)* | *EtOH* | *ced-4(n1162)* | -0.0224 | 0.012 |  | -1.9239 | 0.9026 |  |
| cntrl | *ced-4(n1162)* | *starve* | *ced-4(n1162)* | -0.0646 | 0.016 |  | -40735 | 0.0062 | ** |
