# Supplementary material for: Germ cell apoptosis is critical to maintain Caenorhabditis elegans offspring viability in stressful environments
Source: PLoS One. 2021 Dec 8;16(12):e0260573. doi: 10.1371/journal.pone.0260573 (PMC8654231; doi:10.1371/journal.pone.0260573)
Supplement: S8 Table — Statistical testing for correlations between egg shape (width:length) and egg volume in wild type (N2), ced-9(n1950gf), and germline apoptosis defective (ced-3 and ced-4) mutants after ethanol exposure or starvation. (DOCX) [file pone.0260573.s010.docx]

S8 Tables (accompanies Figure 7B). Statistical testing for correlations between egg shape (width:length) and egg volume in wild type (N2), *ced-9(n1950gf)*, and germline apoptosis defective (*ced-3* and *ced-4*) mutants after ethanol exposure or starvation. Data were fitted to gaussian models (Volume ~ WLratio) for each genotype/environment combination separately with no transformation using the lm function in R. For data representation, see Fig 7B.

Table A. Wildtype-Control

| Source | Estimate | SE | Z-value | Pr(>\|z\|) |  |
| --- | --- | --- | --- | --- | --- |
| Intercept | 3753 | 3572 | 1.051 | 0.296 |  |
| WLRatio | 32496 | 6098 | 5.329 | 5.3E-07 | *** |

Residual std error: 2681 on 110 degrees of freedom

Multiple R-squared 0.2052, **Adjusted R-squared: 0.198**

F-stastistic: 28.4 on 1 and 110 DF, **p-value: 5.29E-07**

Table B. Wildtype-EtOH

| Source | Estimate | SE | Z-value | Pr(>\|z\|) |  |
| --- | --- | --- | --- | --- | --- |
| Intercept | 16625 | 5456 | 3.047 | 0.0031 | ** |
| WLRatio | 9988 | 8766 | 1.139 | 0.2577 |  |

Residual std error: 3934 on 85 degrees of freedom

Multiple R-squared 0.01504, **Adjusted R-squared: 0.003456**

F-stastistic: 1.298 on 1 and 85 DF, **p-value: 0.2577**

Table C. Wildtype-Starvation

| Source | Estimate | SE | Z-value | Pr(>\|z\|) |  |
| --- | --- | --- | --- | --- | --- |
| Intercept | 1761 | 5306 | 0.332 | 0.7407 |  |
| WLRatio | 33449 | 9030 | 3.704 | 0.0004 | *** |

Residual std error: 3630 on 95 degrees of freedom

Multiple R-squared 0.1262, **Adjusted R-squared: 0.117**

F-stastistic: 13.72 on 1 and 95 DF, **p-value: 0.0004**

Table D. *ced-9(n1950gf)-*Control

| Source | Estimate | SE | Z-value | Pr(>\|z\|) |  |
| --- | --- | --- | --- | --- | --- |
| Intercept | 7778 | 6277 | 1.239 | 0.2220 |  |
| WLRatio | 25457 | 10576 | 2.407 | 0.0204 | * |

Residual std error: 3675 on 43 degrees of freedom

Multiple R-squared 0.1188, **Adjusted R-squared: 0.0983**

F-stastistic: 5.794 on 1 and 43 DF, **p-value: 0.02044**

Table E. *ced-9(n1950gf)-*EtOH

| Source | Estimate | SE | Z-value | Pr(>\|z\|) |  |
| --- | --- | --- | --- | --- | --- |
| Intercept | 11569 | 4502 | 2.569 | 0.0143 | * |
| WLRatio | 13395 | 6952 | 1.927 | 0.0617 |  |

Residual std error: 3134 on 37 degrees of freedom

Multiple R-squared 0.0912, **Adjusted R-squared: 0.0666**

F-stastistic: 3.713 on 1 and 37 DF, **p-value: 0.0617**

Table F. *ced-9(n1950gf)-*Starvation

| Source | Estimate | SE | Z-value | Pr(>\|z\|) |  |
| --- | --- | --- | --- | --- | --- |
| Intercept | 12247 | 4879 | 2.510 | 0.0157 | * |
| WLRatio | 13843 | 8015 | 1.727 | 0.0910 |  |

Residual std error: 2658 on 45 degrees of freedom

Multiple R-squared 0.06217, **Adjusted R-squared: 0.04133**

F-stastistic: 2.983 on 1 and 45 DF, **p-value: 0.091**

Table G. *ced-3(n718)-*Control

| Source | Estimate | SE | Z-value | Pr(>\|z\|) |  |
| --- | --- | --- | --- | --- | --- |
| Intercept | 3654 | 4670 | 0.782 | 0.436 |  |
| WLRatio | 33913 | 7752 | 4.375 | 2.82E-05 | *** |

Residual std error: 4068 on 108 degrees of freedom

Multiple R-squared 0.1505, **Adjusted R-squared: 0.1427**

F-stastistic: 19.14 on 1 and 108 DF, **p-value: 2.815E-05**

Table H. *ced-3(n718)-*EtOH

| Source | Estimate | SE | Z-value | Pr(>\|z\|) |  |
| --- | --- | --- | --- | --- | --- |
| Intercept | 15352 | 3676 | 4.177 | 6.25E-05 | *** |
| WLRatio | 7394 | 5671 | 1.304 | 0.195 |  |

Residual std error: 3464 on 102 degrees of freedom

Multiple R-squared 0.01639, **Adjusted R-squared: 0.006747**

F-stastistic: 1on 1 .7 and 102 DF, **p-value: 0.1953**

Table I. *ced-3(n718)-*Starvation

| Source | Estimate | SE | Z-value | Pr(>\|z\|) |  |
| --- | --- | --- | --- | --- | --- |
| Intercept | 20825 | 3974 | 5.24 | 9.51E-07 | *** |
| WLRatio | -4440 | 5618 | -0.79 | 0.431 |  |

Residual std error: 6150 on 96 degrees of freedom

Multiple R-squared 0.006462, **Adjusted R-squared: 0.-0.003887**

F-stastistic: 0.6244 on 1 and 96 DF, **p-value: 0.4314**

Table J. *ced-3(n1286)-*Control

| Source | Estimate | SE | Z-value | Pr(>\|z\|) |  |
| --- | --- | --- | --- | --- | --- |
| Intercept | 16118 | 2776 | 5.807 | 1.91E-07 | *** |
| WLRatio | 11893 | 4370 | 2.721 | 0.00828 | ** |

Residual std error: 2877 on 67 degrees of freedom

Multiple R-squared 0.09952, **Adjusted R-squared: 0.08608**

F-stastistic: 7.405 on 1 and 67 DF, **p-value: 0.008281**

Table K. *ced-3(n1286)-*EtOH

| Source | Estimate | SE | Z-value | Pr(>\|z\|) |  |
| --- | --- | --- | --- | --- | --- |
| Intercept | 5376 | 4626 | 1.162 | 0.25099 |  |
| WLRatio | 25022 | 7505 | 3.334 | 0.00168 | ** |

Residual std error: 2810 on 47 degrees of freedom

Multiple R-squared 0.0.1913, **Adjusted R-squared: 0.1741**

F-stastistic: 11.12 on 1 and 47 DF, **p-value: 0.001677**

Table L. *ced-3(n1286)-*Starvation

| Source | Estimate | SE | Z-value | Pr(>\|z\|) |  |
| --- | --- | --- | --- | --- | --- |
| Intercept | 22928 | 2950 | 7.773 | 6.75E-11 | *** |
| WLRatio | -10347 | 3943 | -2.624 | 0.0108 | * |

Residual std error: 5020 on 66 degrees of freedom

Multiple R-squared 0.09448, **Adjusted R-squared: 0.08076**

F-stastistic: 6.887 on 1 and 66 DF, **p-value: 0.01078**

Table M. *ced-3(n2921)-*Control

| Source | Estimate | SE | Z-value | Pr(>\|z\|) |  |
| --- | --- | --- | --- | --- | --- |
| Intercept | 5885 | 4787 | 1.230 | 0.22324 |  |
| WLRatio | 29334 | 8160 | 3.595 | 0.00062 | *** |

Residual std error: 3015 on 66 degrees of freedom

Multiple R-squared 0.1638, **Adjusted R-squared: 0.1511**

F-stastistic: 12.92 on 1 and 66 DF, **p-value: 0.0006198**

Table N. *ced-3(n2921)-*EtOH

| Source | Estimate | SE | Z-value | Pr(>\|z\|) |  |
| --- | --- | --- | --- | --- | --- |
| Intercept | 25902 | 2463 | 10.516 | <2E-16 | *** |
| WLRatio | -9905 | 3479 | -2.847 | 0.00518 | ** |

Residual std error: 4622 on 122 degrees of freedom

Multiple R-squared 0.06229, **Adjusted R-squared: 0.0546**

F-stastistic: 8.104 on 1 and 122 DF, **p-value: 0.005183**

Table O. *ced-3(n2921)-*Starvation

| Source | Estimate | SE | Z-value | Pr(>\|z\|) |  |
| --- | --- | --- | --- | --- | --- |
| Intercept | 36152 | 2506 | 14424 | <2E-16 | *** |
| WLRatio | -25388 | 3676 | -6.906 | 1.63E-10 | *** |

Residual std error: 5144 on 139 degrees of freedom

Multiple R-squared 0.2555, **Adjusted R-squared: 0.2501**

F-stastistic: 47.7 on 1 and 139 DF, **p-value: 1.632E-10**

Table P. *ced-4(n1162)-*Control

| Source | Estimate | SE | Z-value | Pr(>\|z\|) |  |
| --- | --- | --- | --- | --- | --- |
| Intercept | 7709 | 7101 | 1.086 | 0.28259 |  |
| WLRatio | 34910 | 11772 | 2.965 | 0.00452 | ** |

Residual std error: 4747 on 53 degrees of freedom

Multiple R-squared 0.1423, **Adjusted R-squared: 0.1261**

F-stastistic: 8.794 on 1 and 53 DF, **p-value: 0.004524**

Table Q. *ced-4(n1162)-*EtOH

| Source | Estimate | SE | Z-value | Pr(>\|z\|) |  |
| --- | --- | --- | --- | --- | --- |
| Intercept | 10614 | 5693 | 1.864 | 0.0685 |  |
| WLRatio | 15999 | 9089 | 1.760 | 0.0849 |  |

Residual std error: 4027 on 47 degrees of freedom

Multiple R-squared 0.06184, **Adjusted R-squared: 0.04188**

F-stastistic: 3.098 on 1 and 47 DF, **p-value: 0.08488**

Table R. *ced-4(n1162)-*Starvation

| Source | Estimate | SE | Z-value | Pr(>\|z\|) |  |
| --- | --- | --- | --- | --- | --- |
| Intercept | 23100 | 3843 | 6.012 | 1.06E-07 | *** |
| WLRatio | -5769 | 5695 | -1.013 | 0.315 |  |

Residual std error: 5124 on 62 degrees of freedom

Multiple R-squared 0.01628, **Adjusted R-squared: 0.000415**

F-stastistic: 1.026 on 1 and 62 DF, **p-value: 0.315**
