# Supplementary material for: Germ cell apoptosis is critical to maintain Caenorhabditis elegans offspring viability in stressful environments
Source: PLoS One. 2021 Dec 8;16(12):e0260573. doi: 10.1371/journal.pone.0260573 (PMC8654231; doi:10.1371/journal.pone.0260573)
Supplement: S9 Table — Statistical testing for relationships between egg shape (width:length), egg length, egg width, or egg volume and hatching in ced-3(n718) mutants after starvation treatment. Data were dummy coded and fitted to binomial models as shown below with logistic transformation using the glm function in R. For data representation, see Fig 7C–7F. (DOCX) [file pone.0260573.s011.docx]

S9 Tables (accompanies Figure 7C-F). Statistical testing for relationships between egg shape (width:length), egg length, egg width, or egg volume and hatching in *ced-3(n718)* mutants after starvation treatment. Data were dummy coded and fitted to binomial models as shown below with logistic transformation using the glm function in R. For data representation, see Fig 7C-F.

**Table A. Hatched ~ Volume**

| Source | Estimate | SE | Z-value | Pr(>\|z\|) |  |
| --- | --- | --- | --- | --- | --- |
| Intercept | 1.182 | 1.410 | 0.838 | 0.402 |  |
| Volume | -2.48E-05 | 7.66E-05 | -0.324 | 0.746 |  |

Null deviance: 35.165 on 27 df

Residual deviance: 35.060 on 26 df

**AIC: 39.06**

Number of Fisher Scoring Iterations: 4

**Table B. Hatched ~ Length**

| Source | Estimate | SE | Z-value | Pr(>\|z\|) |  |
| --- | --- | --- | --- | --- | --- |
| Intercept | -10.9274 | 4.4996 | -2.429 | 0.0152 | * |
| Length | 0.3062 | 0.1196 | 2.560 | 0.0105 | * |

Null deviance: 35.165 on 27 df

Residual deviance: 24.135 on 26 df

**AIC: 28.135**

Number of Fisher Scoring Iterations: 5

**Table C. Hatched ~ Width**

| Source | Estimate | SE | Z-value | Pr(>\|z\|) |  |
| --- | --- | --- | --- | --- | --- |
| Intercept | 8.9424 | 4.4119 | 2.027 | 0.0427 | * |
| Width | -0.2807 | 0.1485 | -1.890 | 0.0587 |  |

Null deviance: 35.165 on 27 df

Residual deviance: 30.503 on 26 df

**AIC: 34.503**

Number of Fisher Scoring Iterations: 4

**Table D. Hatched ~ WLRatio**

| Source | Estimate | SE | Z-value | Pr(>\|z\|) |  |
| --- | --- | --- | --- | --- | --- |
| Intercept | 60.77 | 43.22 | 1.406 | 0.160 |  |
| WLRatio | -77.56 | 54.99 | -1.410 | 0.158 |  |

Null deviance: 35.165 on 27 df

Residual deviance: 4.4545 on 26 df

**AIC: 8.4545**

Number of Fisher Scoring Iterations: 9
