# Supplementary material for: Germ cell apoptosis is critical to maintain Caenorhabditis elegans offspring viability in stressful environments
Source: PLoS One. 2021 Dec 8;16(12):e0260573. doi: 10.1371/journal.pone.0260573 (PMC8654231; doi:10.1371/journal.pone.0260573)
Supplement: S10 Table — Statistical testing for differences in embryo production in wild type (N2) versus apoptotic defective (ced-3) mutants after acid or ethanol exposure, oxidative stress, or starvation. (DOCX) [file pone.0260573.s012.docx]

S10 Tables (accompanies S1 Figure). Statistical testing for differences in embryo production in wild type (N2) versus apoptotic defective (*ced-3*) mutants after acid or ethanol exposure, oxidative stress, or starvation.

Data were fitted to negative binomial models (Total embryos ~ Genotype + Environment) with log transformation and overdispersion parameters of 6.61 and 12, respectively (A, D). The R software package ‘Dharma’ was used to evaluate the models. Models did not perform significantly better if interactions were included. Therefore, we modeled only main effects. The R software package, ‘emmeans’ was used to obtain estimated marginal means on the response scale (B, E) and contrasts (C, F) with Tukey corrected p-values. For data representation, see S1 Figure.

Table A. Total embryos laid after acid or oxidative stress: Conditional model

| Source | Estimate | SE | Z-value | Pr(>\|z\|) |  |
| --- | --- | --- | --- | --- | --- |
| Intercept | 5.60506 | 0.0367 | 152.74 | <2E-16 | *** |
| Geno ced-3 | -0.33683 | 0.04022 | -8.37 | <2E-16 | *** |
| Env HCl | -0.31176 | 0.04726 | -6.6 | 4.19E-11 | *** |
| Env paraquat | -0.3538 | 0.04936 | -7.17 | 7.63E-13 | *** |

**Table B. Total embryos laid after acid or oxidative stress: Emmeans**

| Genotype | Environment | response | SE | df |
| --- | --- | --- | --- | --- |
| N2 | *control* | 271.798573 | 9.97381815 | 97 |
| N2 | *HCl* | 198.998345 | 7.62545102 | 97 |
| N2 | *paraquat* | 190.806893 | 7.8110743 | 97 |
| ced-3 | *control* | 194.071877 | 7.88501177 | 97 |
| ced-3 | *HCl* | 142.090453 | 5.82233257 | 97 |
| ced-3 | *paraquat* | 136.241524 | 5.90485031 | 97 |

**Table C. Total embryos laid after acid or oxidative stress: Contrasts**

| Env1 | Geno1 | Env2 | Geno2 | ratio | SE | df | t-ratio | p-value |  |
| --- | --- | --- | --- | --- | --- | --- | --- | --- | --- |
| control | *N2* | *HCl* | *N2* | 1.3658 | 0.0645 | 97 | 6.5970 | 3.34E-08 | *** |
| control | *N2* | *paraquat* | *N2* | 1.4245 | 0.0703 | 97 | 7.1676 | 2.63E-09 | *** |
| control | *ced-3* | *HCl* | *ced-3* | 1.3658 | 0.0645 | 97 | 6.5970 | 3.34E-08 | *** |
| control | *ced-3* | *paraquat* | *ced-3* | 1.4245 | 0.0703 | 97 | 7.1676 | 2.63E-09 | *** |
| control | *N2* | *control* | *ced-3* | 1.4005 | 0.0563 | 97 | 8.3749 | 3.83E-10 | *** |
| HCl | *N2* | *HCl* | *ced-3* | 1.4005 | 0.0563 | 97 | 8.3749 | 3.83E-10 | *** |
| paraquat | *N2* | *paraquat* | *ced-3* | 1.4005 | 0.0563 | 97 | 8.3749 | 3.83E-10 | *** |

Table D. Total embryos laid after ethanol or starvation: Conditional model

| Source | Estimate | SE | Z-value | Pr(>\|z\|) |  |
| --- | --- | --- | --- | --- | --- |
| Intercept | 5.46215 | 0.04731 | 115.45 | <2E-16 | *** |
| Geno ced-3 | -0.28926 | 0.05703 | -5.07 | 3.93E-07 | *** |
| Env EtOH | -0.29756 | 0.06602 | -4.51 | 6.57E-06 | *** |
| Env starved | -0.62845 | 0.07107 | -8.84 | <2E-16 | *** |

**Table E. Total embryos laid after ethanol or starvation: Emmeans**

| Genotype | Environment | response | SE | df |
| --- | --- | --- | --- | --- |
| control | *N2* | 235.603672 | 11.1471552 | 95 |
| EtOH | *N2* | 174.965828 | 9.99028071 | 95 |
| starved | *N2* | 125.675843 | 7.89758858 | 95 |
| control | *ced-3* | 176.423446 | 9.27733289 | 95 |
| EtOH | *ced-3* | 131.016949 | 8.16884163 | 95 |
| starved | *ced-3* | 94.1078933 | 6.40435622 | 95 |

**Table F. Total embryos laid after ethanol or starvation: Contrasts**

| Env1 | Geno1 | Env2 | Geno2 | ratio | SE | df | t-ratio | p-value |  |
| --- | --- | --- | --- | --- | --- | --- | --- | --- | --- |
| control | *N2* | *EtOH* | *N2* | 1.3466 | 0.0889 | 95 | 4.5073 | 2.65E-04 | *** |
| control | *N2* | *starved* | *N2* | 1.8747 | 0.1332 | 95 | 8.8432 | 4.64E-10 | *** |
| control | *ced-3* | *EtOH* | *ced-3* | 1.3466 | 0.0889 | 95 | 4.5073 | 2.65E-04 | *** |
| control | *ced-3* | *starved* | *ced-3* | 1.8747 | 0.1332 | 95 | 8.8432 | 4.64E-10 | *** |
| control | *N2* | *control* | *ced-3* | 1.3354 | 0.0762 | 95 | 5.0721 | 2.83E-05 | *** |
| EtOH | *N2* | *EtOH* | *ced-3* | 1.3354 | 0.0762 | 95 | 5.0721 | 2.83E-05 | *** |
| starved | *N2* | *starved* | *ced-3* | 1.3354 | 0.0762 | 95 | 5.0721 | 2.83E-05 | *** |
